# Hybrid real-synthetic dataset framework for robotic hazard detection in industrial environments

**DOI:** 10.1038/s41598-025-33603-5

**Published:** 2026-01-12

**Authors:** Amr Khamis, Heba A. Shaban, Heba A. Fayed, Moustafa H. Aly

**Affiliations:** Department of Electronics and Communications Engineering, College of Engineering and Technology, Arab Academy for Science, Technology & Maritime Transport, Alexandria, 1029 Egypt

**Keywords:** Engineering, Mathematics and computing

## Abstract

The increasing complexity of industrial environments requires the development of real-time hazard detection and environmental monitoring using intelligent robotic systems. This paper introduces *RoboFusion*, an integrated framework that combines Autonomous Mobile Robots (AMRs), fixed sensing nodes, and a novel hybrid dataset generation pipeline for data-driven industrial safety. Deployed in a functioning industrial testbed, *RoboFusion* collected real-time telemetry over 180 days using four sensor suites: two fixed units and two additional units mounted on Near-Field Communication (NFC) guided AMRs, each equipped with 12 sensors sampling at one-minute intervals. This deployment yielded approximately one million multi-modal sensor records, including temperature, humidity, gas concentrations, air quality, and pressure. Data streams were processed onboard using ESP32 microcontrollers, and they were transmitted via Message Queuing Telemetry Transport (MQTT) to an Internet of Things (IoT) cloud platform. The scarcity and imbalance of hazard events in real collected data create a challenge for effective model training. *RoboFusion* addresses this issue through a structured synthetic dataset generation framework. This framework augments non-hazardous data using statistical augmentation techniques, and it simulates hazardous data through multi-phase curve fitting, spatial propagation modeling, and location-aware hazard scenarios. The resulting synthetic dataset improves coverage of rare and safety-critical scenarios while maintaining consistency with real-world dynamics. Evaluation across four machine learning models, namely Random Forest (RF), Support Vector Machine (SVM), Extreme Gradient Boosting (XGBoost), and Multi-Layer Perceptron (MLP), demonstrates significant cross-domain gains. As an example, hazard F1 scores improved from 0.47 to 0.85 for the RF model, and from 0.16 to 0.79 for the SVM model when models trained on synthetic data were tested against real hazard events. *RoboFusion* therefore delivers a reproducible robotic sensing platform and an openly accessible hybrid dataset. It introduces a novel approach to hazard simulation that mimics real-world hazards and supports the development of resilient Artificial Intelligence (AI) systems for industrial hazard detection and autonomous safety intelligence.

## Introduction

Modern industrial environments are becoming increasingly complex, demanding advanced, real-time solutions to enhance operational efficiency, situational awareness, and safety. In response, industries are adopting Autonomous Mobile Robots (AMRs), which represent a significant evolution from traditional Automated Guided Vehicles (AGVs) that rely on static paths and complex infrastructure. AMRs can autonomously navigate, execute logistics tasks, and collect telemetry data, and they serve as key enablers of Industry 4.0 in smart manufacturing systems^1^.

The capabilities of AMRs have grown substantially with the integration of multi-sensor systems, Internet of Things (IoT) communication protocols, and Artificial Intelligence (AI) driven analytics. These systems can detect critical environmental anomalies such as gas leaks, temperature spikes, fire hazards, and airborne pollutants^2^. However, the reliability of AI-based hazard detection in industrial contexts is often limited by the scarcity of high-quality datasets, particularly those capturing rare and unpredictable hazard scenarios. Simulating hazardous events in real industrial facilities is constrained by safety, ethical, and regulatory concerns. Furthermore, environmental variability across time and space creates a need for datasets that are both statistically robust and operationally diverse^3^. Conventional hazard detection systems, which typically rely on fixed sensor networks, lack the mobility and adaptability required for effective deployment in large industrial spaces. This limitation leads to biased learning models, higher false negatives, and poor generalization when the system encounters unseen anomalies^4^.

Internet of Robotic Things (IoRT) enables robotic systems to sense, interpret, and respond in real time. In *RoboFusion* this is achieved through embedded edge computing on ESP32 microcontrollers and through Message Queuing Telemetry Transport (MQTT) protocol based communication, which together provide low-latency real-time data processing onboard the AMRs^5^. This design minimizes reliance on cloud services, reduces latency^6,7^, and supports predictive modeling to enhance system resilience and reduce operational downtime^8^.

While the hardware platform in *RoboFusion*, which features a vision-free Near-Field Communication (NFC) guided ESP32 based dual-AMR system and four sensor suites, two mobile and two fixed, is essential for comprehensive real data collection, the core novelty of *RoboFusion* lies in its synthetic dataset generation framework. *RoboFusion* introduces a dual-pipeline approach that transforms real, multi-modal, multi-location sensor data into a comprehensive synthetic dataset that mirrors real-world behavior.

Specifically, *RoboFusion* addresses the critical scarcity of hazard data by:

1. Collecting real-world data (180 days at a one-minute sampling rate) from AMRs and fixed sensor suites, covering both normal operations and rare hazard events, and using this as the foundational dataset.
2. Augmenting real normal data to generate synthetic normal data via noise injection, time warping, and feature scaling, thereby reflecting the natural variability of industrial processes.
3. Generating synthetic hazard data through a multi-phase modeling process that fits mathematical functions to real hazard events (covering the ignition, growth, peak, and decay stages), simulates spatial propagation, and produces diverse, location-aware hazard scenarios.

*RoboFusion* distinguishes itself from prior works by:

1. Integrating multi-modal data from 12 distinct sensors across four sensor suites (two mobile AMRs and two fixed stations) within a real industrial facility.
2. Generating the first hybrid dataset that combines real and synthetic data for industrial hazard detection, validated across multiple machine learning models.
3. Providing a scalable, reproducible, and open-access dataset that supports research in AI-driven robotics, environmental monitoring, and industrial safety.

The remainder of this paper is organized as follows: Section "Background and related work" reviews background and related work. Section “System architecture” outlines the system architecture. Section “Methods” details the *RoboFusion* methodology. Section “Results” presents results and validation. Section “Discussion” discusses findings, and Section “Conclusions” concludes with future directions.

## Background and related work

### Autonomous and guided mobile robots in industrial sensing

Recent studies on Autonomous Mobile Robots (AMRs) and Automated Guided Vehicles (AGVs) for industrial automation^5–16^ have explored Light Detection and Ranging (LiDAR) and Inertial Measurement Unit (IMU) based navigation, Reinforcement Learning (RL), and Convolutional Neural Network (CNN) driven obstacle avoidance. However, these approaches typically require high computational power, dense training data, and centralized control architectures. In contrast, *RoboFusion* employs a lightweight Near Field Communication (NFC) guided navigation system combined with decentralized edge processing, which enables reliable real-time operation in an industrial testbed.

While simulation-based RL approaches for AMR navigation were demonstrated in^9^ and Long Short-Term Memory (LSTM) based obstacle avoidance was proposed in^7^, these techniques faced high data requirements. Similarly, CNN driven vision systems^10^ were limited in low-light environments. Earlier AGV frameworks^11–15^ rely on centralized or semi-centralized control and communication improvement strategies, whereas *RoboFusion* operates fully on embedded controllers with both mobile and fixed sensing nodes to achieve distributed, low-latency decision-making.

Unlike anomaly detection systems that rely solely on internal telemetry such as motor current or velocity^16^, RoboFusion captures the environmental context, a capability that enables synthetic hazard generation to expand model robustness under realistic industrial safety conditions.

### Internet of Robotic Things (IoRT)

The Internet of Robotic Things (IoRT) extends the Internet of Things (IoT) paradigm by integrating robotic systems with cloud and edge analytics for adaptive and connected automation^17–27^. IoRT applications span multiple domains, including secure robotic healthcare frameworks^17^, industrial manufacturing^18,19^, and predictive maintenance^20–22^. Although these systems have advanced connectivity, they often face latency and heterogeneity challenges. Vision based IoRT systems using Simultaneous Localization and Mapping (SLAM)^23,24^ improved perception and autonomous mapping, although they remain computationally expensive.

*RoboFusion* instead achieves IoRT functionality through embedded microcontrollers and lightweight Message Queuing Telemetry Transport (MQTT) protocols, providing low-latency and bidirectional communication between mobile robots, fixed sensing nodes, and cloud dashboards. While theoretical IoRT architectures were explored in^25–27^, RoboFusion demonstrates a fully implemented and hardware integrated deployment validated in a real industrial facility, which bridges the gap between conceptual models and operational systems.

### Sensor fusion and synthetic datasets

Sensor fusion remains central to robotic perception and localization, traditionally combining LiDAR, IMU, and odometry data^28,29^. *RoboFusion*, however, collects twelve heterogeneous sensory inputs, including temperature, humidity, pressure, air quality, and multiple gas concentrations, to form high-dimensional hazard profiles. Although no explicit fusion algorithm is applied, the framework’s multi-modal data architecture enables downstream machine learning for anomaly and hazard detection.

Data augmentation for robust sensing and modeling under noise has been studied in^30–34^, including smartphone based telemetry^31^, time series augmentation^32,33^, and domain adaptation^34^. Building upon these foundations, *RoboFusion* introduces a dual-pipeline synthetic data generation framework that augments normal operating data and simulates hazard events through phase segmentation, curve fitting, normalization, and spatial scaling. The resulting hybrid real and synthetic dataset provides balanced class representation and greater diversity for AI based hazard prediction, capabilities that were not previously demonstrated in industrial robotics datasets.

### Environmental and gas hazard monitoring

Indoor environmental and air quality monitoring has been widely studied across diverse contexts, including urban comfort modeling^35–38^, thermal environments^39–42^, and large-scale building datasets^43–49^. However, these studies primarily rely on fixed sensors and lack mobile data collection. *RoboFusion* contributes the first combined real and synthetic industrial dataset for Egypt, covering both Mediterranean and desert climatic conditions and integrating mobile and fixed sensing modalities for continuous environmental coverage. Further, large-scale air quality datasets and pollutant tracking efforts^50–54^, Heating, Ventilation, and Air Conditioning (HVAC) adaptation systems^55–57^, and simulation-based datasets^57,58^ provide valuable context but lack the dynamic, multi-robot sensing found in *RoboFusion*. IoT-enabled monitoring platforms for industrial and domestic use^59–61^ demonstrate data integration capabilities, though without mobile sensing for real-time hazard localization.

Gas hazard detection research has employed LiDAR-based mapping^62^, threshold triggers^63^, and Receding Horizon Control (RHC) for gas leak mitigation^64^, while swarm-based detection with nano quadcopters was proposed in^65^. Other studies explored Robot Operating System (ROS)-based frameworks^66^, optical spectroscopy for gas leak sensing^66^, and digital-twin-based predictive maintenance^67,68^. Unlike these simulation-oriented systems, *RoboFusion* fuses real-world sensor data with structured synthetic generation, enabling machine-learning–ready hazard prediction under realistic conditions. Finally, by combining low-cost sensors, edge computation, and modular mobile platforms, *RoboFusion* achieves scalable, cost-efficient environmental monitoring and hazard detection. A comparative analysis of all related environmental datasets, sensing domains, and machine learning applications is provided in Supplementary Appendix B (Tables B1 and B2).

## System architecture

*RoboFusion* is a decentralized robotic sensing platform designed to collect high-resolution, multi-modal environmental data from industrial facilities. Its core architecture enables both real-time hazard detection and the generation of a hybrid dataset that combines real-world observations with synthetic scenarios for machine learning applications.

**Fig. 1:**
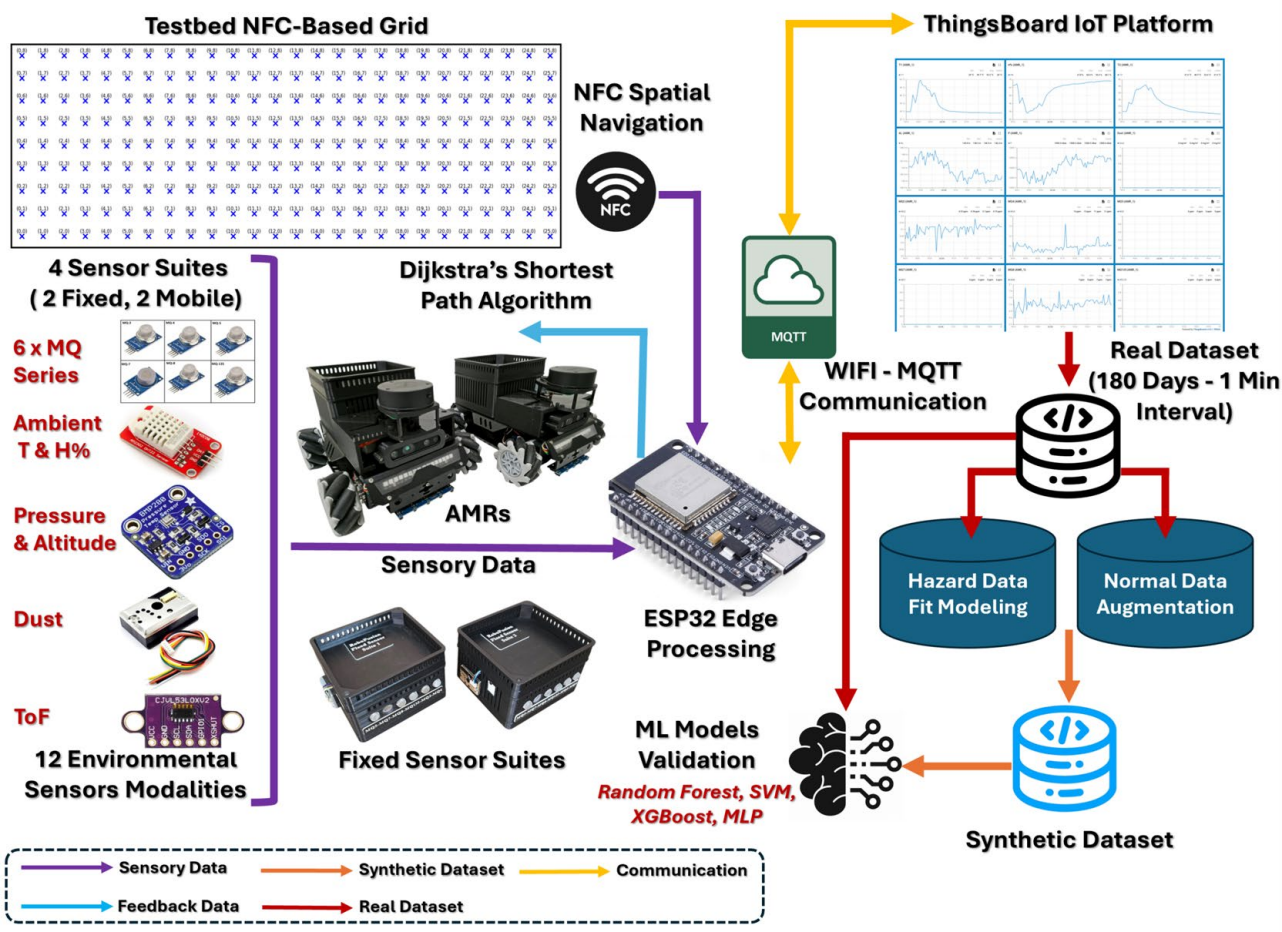
*RoboFusion* system end to end block diagram.

The system consists of four sensor suites, two are mounted on Autonomous Mobile Robots ($\hbox {AMR}_1$ and $\hbox {AMR}_2$) and two fixed sensor suites ($\hbox {Suite}_1$ and $\hbox {Suite}_2$), equipped with identical sensor arrays deployed at strategic locations within a 9 m $\times$ 26 m industrial testbed at the Industry Service Complex (ISC) of Arab Academy for Science, Technology and Maritime Transport (AASTMT) main campus, Alexandria, Egypt. The testbed is segmented into 200 one-square-meter zones, each marked by an NFC tag (Type A, 13.56 MHz) adhered to the floor. These tags act as static beacons for AMR localization, enabling a grid-based navigation strategy. The overall system block diagram is illustrated in Fig. 1.

### Sensor suites

Each sensor suite integrates a 12-sensor array designed for multi-modal environmental monitoring, including both physical and chemical measurements. As shown in Table 1, each suite comprises four domains: (1) Ambient sensing is handled by the DHT-22 for ambient temperature (T1) and humidity (H%), (2) Onboard telemetry is measured by the BMP280 sensor, which provides pressure (P), altitude (AL), and onboard temperature (T2), (3) Particulate matter concentration is detected using the GP2Y1010AU0F optical dust sensor, and (4) Gas detection is performed using six analog MQ sensors (MQ2, MQ4, MQ5, MQ7, MQ8, MQ135) capable of sensing a range of hazardous gases (e.g., LPG, methane, CO, $\hbox {H}_2$, VOCs) and measured in parts per million (ppm).

**Fig. 2:**
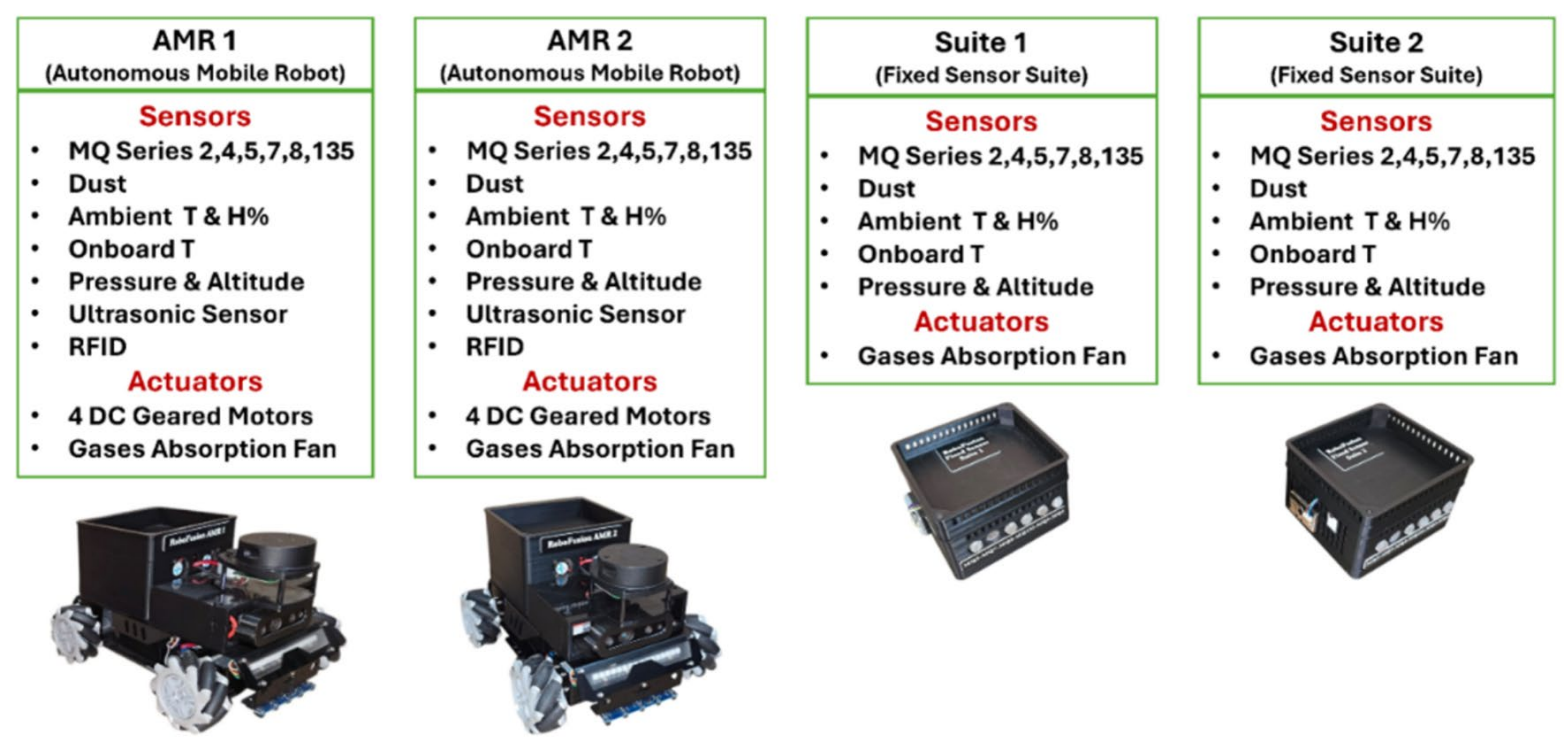
Sensor suites composition overview.

**Fig. 3:**
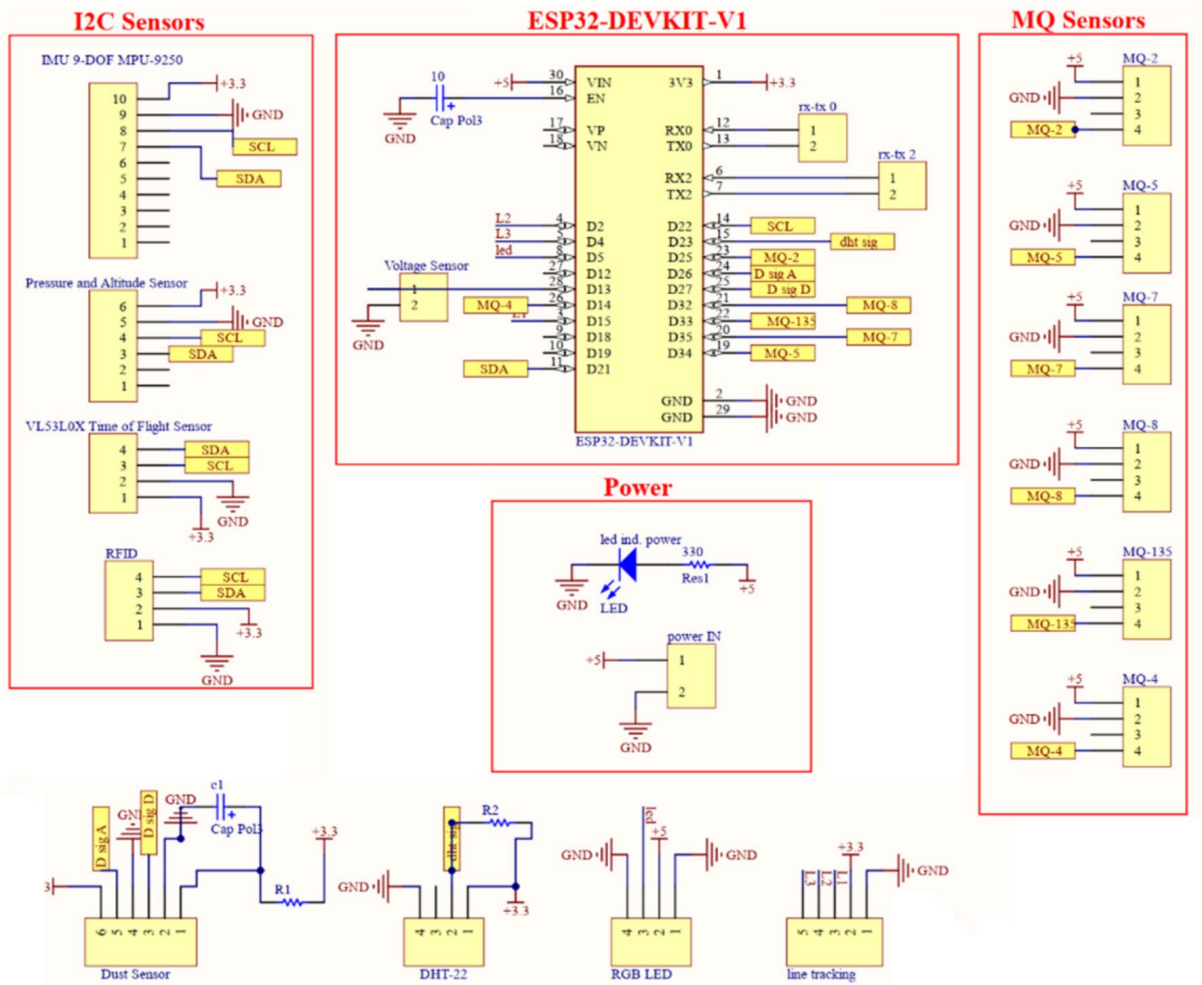
Hardware schematic of the *RoboFusion* sensing module (created by the authors using Altium Designer).

To ensure consistent air sampling across sensor surfaces, particularly in areas of low circulation, each suite includes a Gas Exchange Assist Fan (GEAF), which is a 5,Volts Direct Current (VDC) axial fan operating at 4000,Revolutions Per Minute (RPM). This active airflow system enhances the accuracy and stability of gas concentration sensor readings. The BMP280 sensor is intentionally placed near the ESP32 board to serve as a thermal reference that distinguishes environmental heating from microcontroller-induced heat, while the DHT-22 provides external thermal measurements. The layout of the four sensor suites is shown in Fig. 2, and their internal schematic diagram is represented in Fig. 3.

**Table 1:** Sensor suites composition.

**Domain**	**Sensor**	**Measurand**	**Interface**	**Use Case**
Ambient	DHT-22	Ambient temperature (T1), humidity (H%)	Digital (single-wire)	General indoor ventilation monitoring
Telemetry (Onboard)	BMP280	Pressure (P), altitude (AL), onboard temperature (T2)	I^2^C	Fire-risk detection (pressure drop), gas-dispersion estimation
Particulate Matter	GP2Y1010AU0F	Dust concentration (ppm)	Analog	Smoke, dust and particulate detection
Gas Detection	MQ2, MQ4, MQ5, MQ7, MQ8, MQ135	Gas concentrations (ppm): LPG, methane, CO, H_2_, VOCs, etc.	Analog	Fire hazards, gas leaks and chemical-anomaly detection

### Autonomous Mobile Robots ($\hbox {AMR}_1$ and $\hbox {AMR}_2$)

**Fig. 4:**
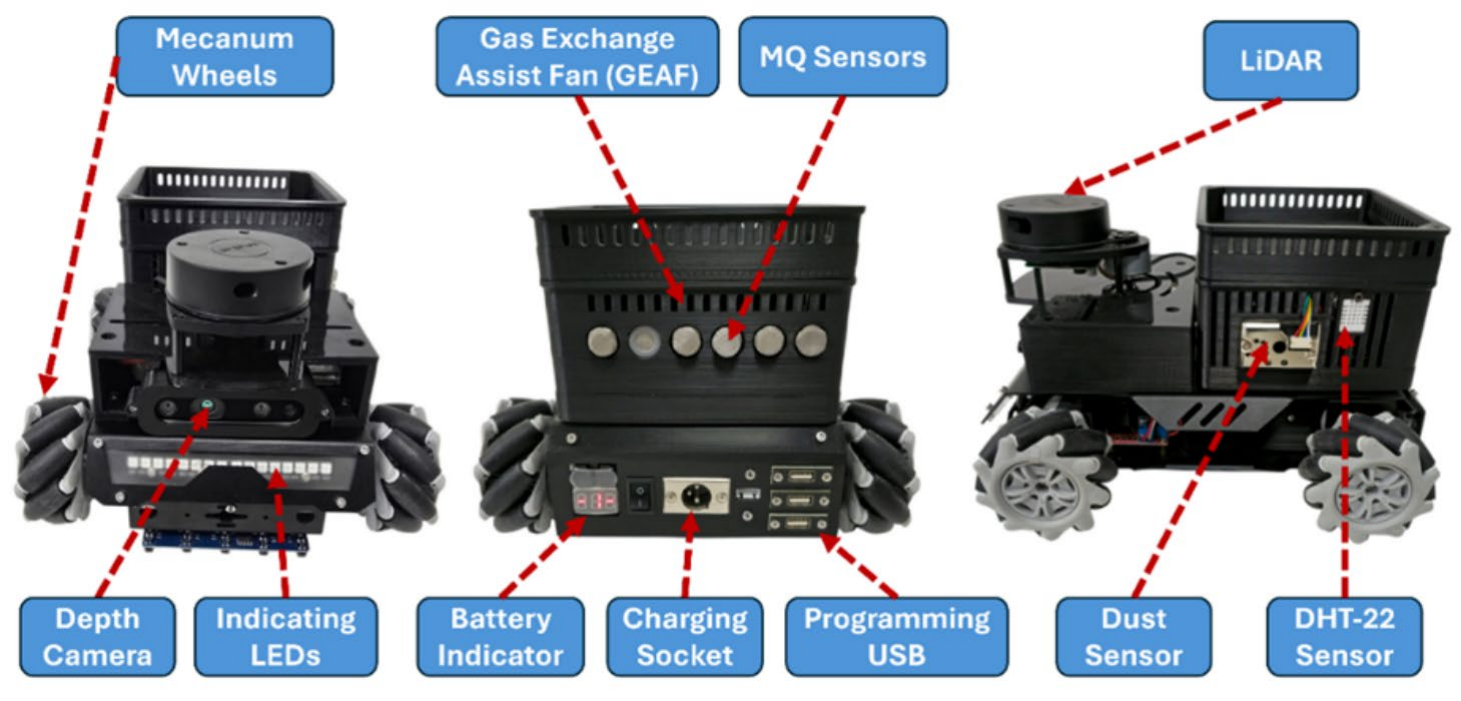
AMRs front and back views – main components.

The AMRs serve as mobile sensor platforms capable of navigating autonomously across the testbed environment along either predefined or dynamically generated patrol routes. As shown in Fig. 4, each AMR is constructed on a custom-designed omnidirectional chassis measuring 285 mm $\times$ 250 mm and fitted with four 60 mm Mecanum wheels. This wheel configuration allows the robot to maneuver in any direction without changing orientation, a feature that enables precise navigation in the grid-constrained layout of the testbed. Each AMR is actuated by four GM37-520 DC gear motors (12 V, 152 RPM, 7 kg$\cdot$cm torque), each equipped with Hall-effect encoders providing 11 pulses per revolution (PPR). The encoder signals are processed using interrupt-driven counters on the ESP32, which estimates rotational speed and computes linear displacement using wheel diameter and gear ratios.

**Fig. 5:**
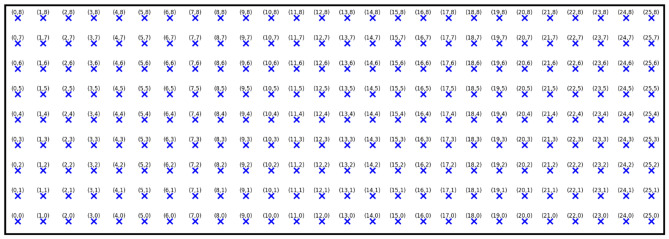
Testbed grid configuration.

Position tracking is achieved using a hybrid localization strategy. At each grid intersection, an NFC reader attached to the robot detects passive tags embedded in the floor. These tags correspond to unique cell identifiers (C00 to C199), mapped to (x, y) coordinates via an onboard lookup table where $x = [0-8]$ and $y = [0-25]$. Between tags, dead-reckoning is applied using encoder feedback to maintain positional continuity. The grid layout and tag positions are illustrated in Fig. 5.

Navigation follows a rule-based logic with fallback mechanisms. A custom implementation of Dijkstra’s Shortest Path Algorithm computes optimal routes based on Euclidean distance between Cells. During traversal, the robot validates cell transitions through combined encoder data and tag recognition.

To avoid collisions, each AMR is equipped with three VL53L0X Time-of-Flight (ToF) sensors mounted at the front and sides, with a maximum range of 200 cm and an obstacle threshold of 3 cm. The chassis is fabricated from lightweight aluminum alloy with vibration damping and a low center of gravity, supporting a payload capacity of up to 25 kg. Each robot is powered by an 11.1 V, 2200 mAh Lithium-ion (Li-ion) battery pack, offering approximately 2 hours of operation under combined sensing and mobility workloads.

### Embedded firmware and data flow

The ESP32 firmware manages all sensing, localization, data processing, and wireless communication. Analog gas sensor readings are converted into parts per million (ppm) values using manufacturer-provided sensitivity curves from datasheets and empirical calibration performed during lab-based gas exposure experiments. Digital sensors are interfaced through designated General-Purpose Input/Output (GPIO) or Inter-Integrated Circuit (I^2^C) lines. For example, the DHT-22 sensor operates on GPIO4 with a 10 k$\Omega$ pull-up resistor, while the BMP280 communicates via I^2^C using GPIO21 (Serial Data (SDA)) and GPIO22 (Serial Clock (SCL)).

The adopted sampling rate of one reading per minute was selected after testing higher rates (2 s and 10 s), which produced denser data without additional informative variation. This rate therefore offered a practical balance between temporal resolution and system stability during the 180-day deployment. Nevertheless, the *RoboFusion* framework remains fully programmable, allowing adaptive or higher-frequency sampling when required by specific test conditions.

The firmware acquires sensor data at each sampling interval, performs NFC-based zone identification, applies calibration functions, and formats telemetry into structured JavaScript Object Notation (JSON) messages. These packets include suite ID, coordinates, sensor readings, and a timestamp generated from the ESP32 system clock. If Wi-Fi connectivity is lost, data is cached in flash memory and retransmitted once the connection is restored. The telemetry schema and communication logic are designed to be modular, allowing additional sensor suites or AMRs to be added without modifying existing infrastructure.

### Communication and cloud integration

All sensor suites transmit their telemetry via the MQTT protocol to the ThingsBoard Cloud platform. Each suite publishes a unique topic (for example, telemetry/AMR1 or telemetry/Suite2), and messages are formatted in JSON to maintain compatibility with cloud based analytics tools. MQTT communication occurs over port 1883 with Wi-Fi Protected Access 2 using a Pre Shared Key (WPA2 PSK) secured Wireless Fidelity (Wi-Fi) connection. Static Internet Protocol (IP) addressing ensures deterministic routing and device identification.

**Fig. 6:**
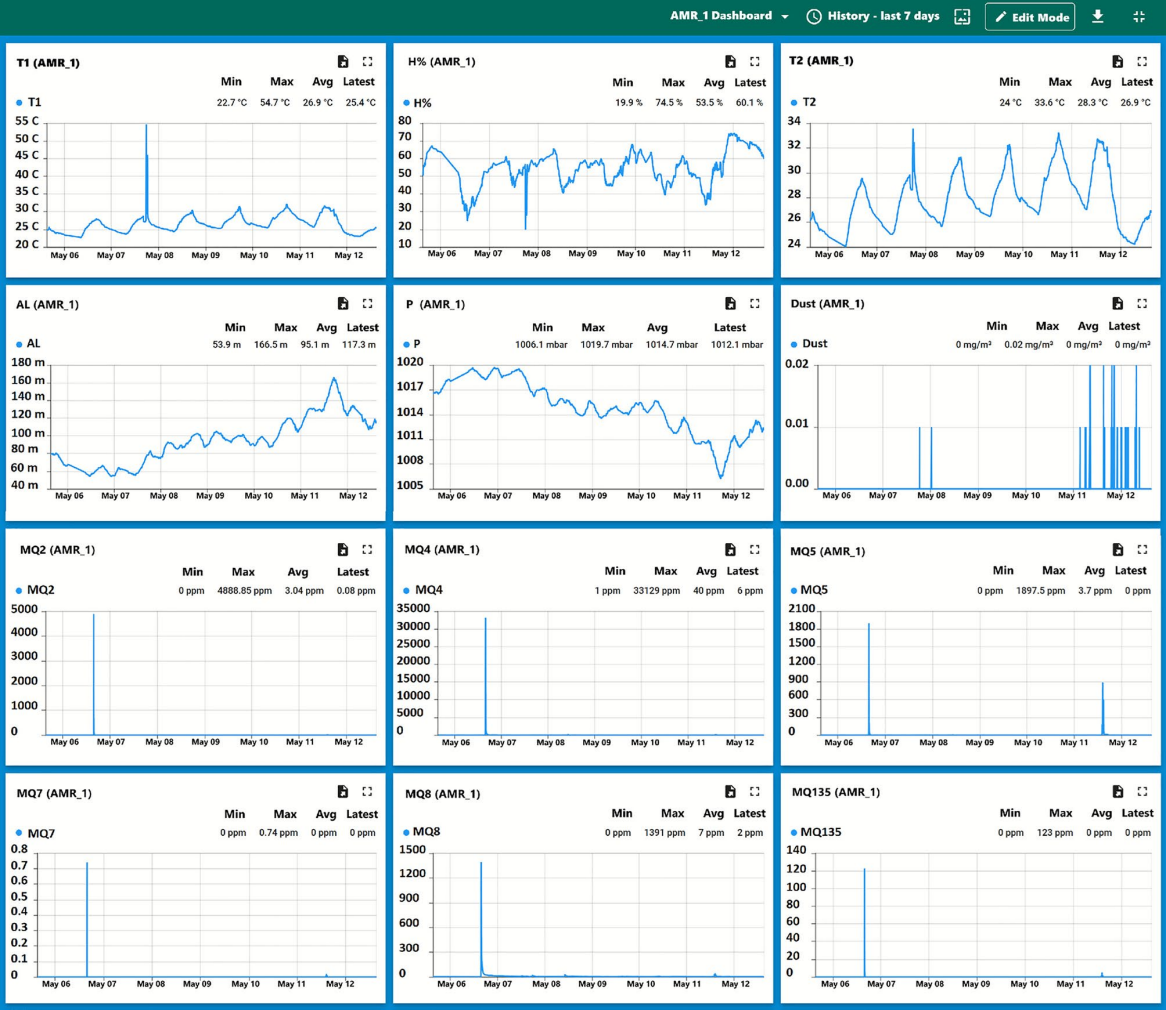
Dashboard Sample for $\hbox {AMR}_1$ displaying the reading of 12 sensors array.

ThingsBoard platform supports real-time dashboards as shown in Fig. 6, rule-based alerts, device telemetry, and remote procedure calls (RPCs). Telemetry can be exported using the built-in utilities in Comma-Separated Values (CSV)/Excel formats, each containing 15 fields: Date, Time, T1, H%, P, AL, T2, Dust, MQ2, MQ4, MQ5, MQ7, MQ8, MQ135, and Condition.

### Summary and design rationale

The *RoboFusion* system is tailored to operate under realistic industrial conditions, where infrastructure may be limited and operational safety is critical. NFC based localization eliminates the need for power intensive visual navigation. Modular firmware and a decentralized architecture ensure resilience and scalability. Preprocessing steps, including analog calibration, zone tagging, and fault handling, are managed locally at the edge, which reduces latency and minimizes dependency on cloud services. By combining autonomous mobile sensing, robust embedded processing, and scalable cloud integration, *RoboFusion* provides a reliable platform for real-time industrial monitoring and hybrid dataset creation. Its use of low-cost hardware and communication protocols ensures accessibility for research groups aiming to replicate or extend the system.

Additional implementation-level details related to the firmware architecture and system deployment are provided in Appendix C. In particular, the end-to-end sensing, communication, and cloud integration pipeline is illustrated in Supplementary Figure C1.

## Methods

The *RoboFusion* framework follows a structured and reproducible methodology designed to generate hybrid environmental datasets for training AI based hazard detection models in industrial robotic environments. The primary aim is to simulate both normal and hazard operational scenarios that can arise in dynamic industrial settings. This framework enables learning algorithms to generalize effectively, especially when deployed on autonomous robotic platforms.

**Fig. 7:**
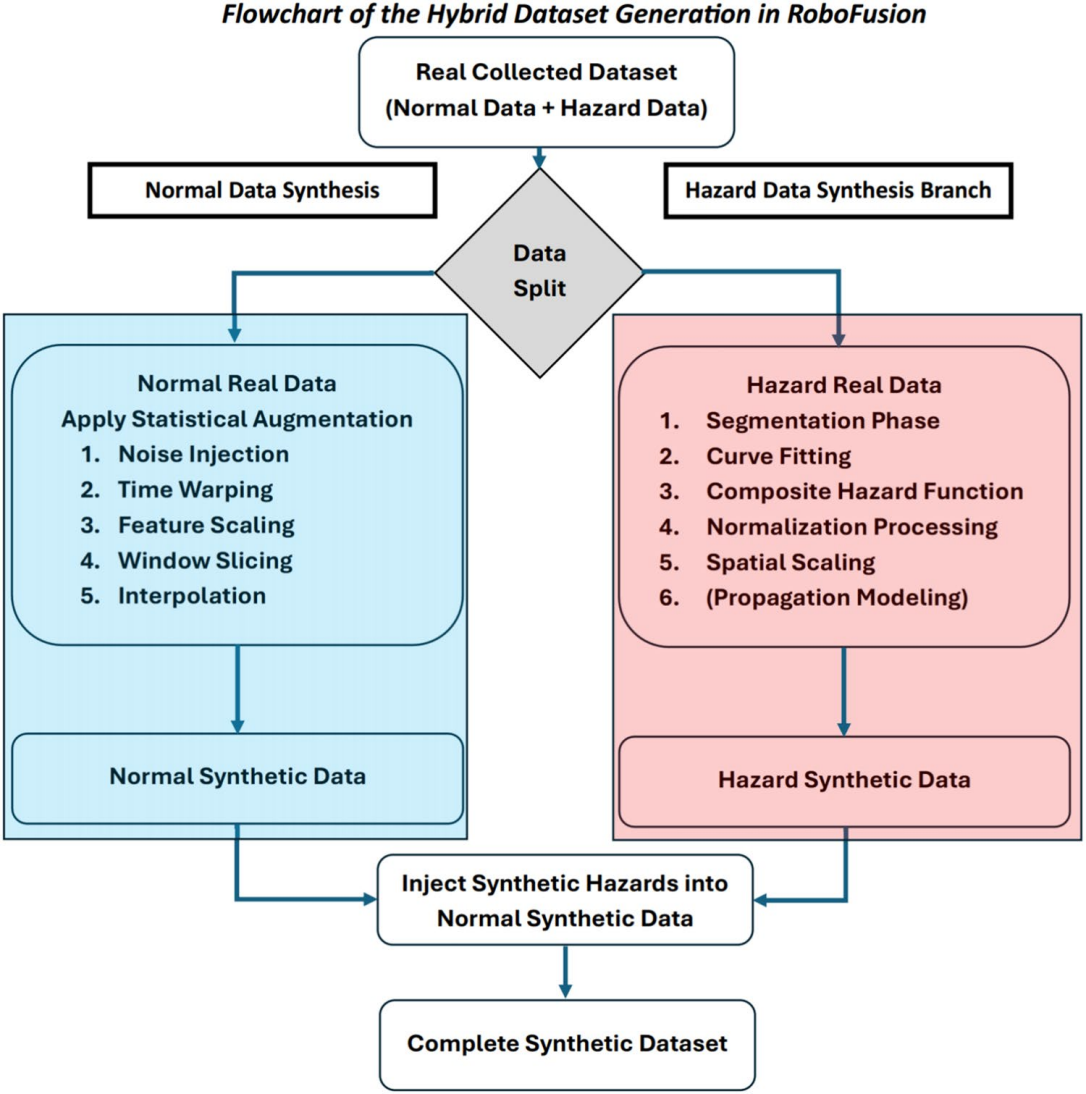
Overview of the *RoboFusion* dataset generation pipeline, including real-world data acquisition, synthetic normal and hazard data creation, and hazard injection.

At the core of this methodology is a hybrid sensing system that combines real sensor data, collected from the four sensor suites, with a dual synthetic data generation pipeline. This architecture as shown in Fig. 7, addresses a fundamental limitation in industrial robotics research, which is the scarcity of high fidelity hazard data due to the rarity, unpredictability, and safety risks associated with such events.

The methodology comprises three main phases:

1. Real-world data acquisition, performed over a 180-day deployment across the four distributed sensor suites.
2. Synthetic data generation, using independent branches for normal condition augmentation and hazard condition modeling.
3. Hybrid dataset assembly, in which generated synthetic hazards are injected into dense synthetic normal data at semantically meaningful intervals and spatial positions, forming a continuous, labeled time series for machine learning applications.

This process yields a robust dataset that not only captures the complex dynamics of industrial hazards but also enables mobile robotic platforms to be trained under diverse environmental conditions.

### Real dataset collection

Environmental sensing data was collected and transmitted wirelessly via Wi-Fi to a central router, then forwarded to the ThingsBoard IoT platform. Each suite was registered as a separate IoT device with unique tokens. The real dataset pipeline in *RoboFusion* begins with exporting environmental telemetry data from the ThingsBoard cloud platform, where each of the four sensor suites generates 12 separate CSV files corresponding to individual sensor channels.

This results in 48 timestamped files containing raw sensor readings. These files are then merged using a Python script into a unified dataset by aligning records through a common timestamp key and adding a new column namely “Suite_ID” to distinguish the origin of each reading.

Following this, interpolation is applied to compensate for data losses caused by network interruptions, ensuring temporal continuity in the dataset. The interpolated dataset is subsequently used to generate a set of 12 time-series plots each corresponding to one sensor modality where sensor outputs from all four suites are color-coded and displayed together to allow for comparative analysis across space and time.

**Fig. 8:**
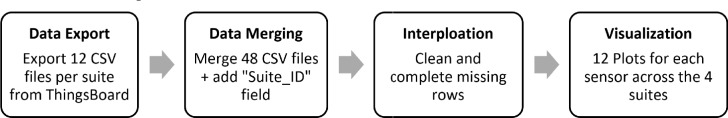
Workflow of *RoboFusion* real dataset processing: From telemetry export on the ThingsBoard platform to dataset consolidation, interpolation, and multi-suite visualization.

This streamlined data pipeline, shown in Fig. 8 transforms raw telemetry into a structured, complete, and interpretable real-world dataset. The complete schema of the real dataset is illustrated in Table 2.

**Table 2:** Real dataset schema.

Date	Time	Suite_ID	T1	T2	H%	P	AL	Dust	MQ2	MQ4	MQ5	MQ7	MQ8	MQ135	Condition
Schema (column names) only; values omitted for brevity.															

The testbed hosted 15 controlled real hazard events of three types, (1) five fire events (labeled as Hazard_1), (2) four high-temperature fluctuations events (labeled as Hazard_2), and (3) six gas leaks events (labeled as Hazard_3). Each event lasted between 30–120 minutes, yielding labeled sequences for supervised learning. Each hazard scenario was carefully carried out under safe, controlled conditions, following AASTMT safety guidelines. Trained staff supervised the experiments, and risk assessments were completed beforehand. During data collection, all necessary precautions like wearing wearing Personal Protective Equipment (PPE), keeping safe distances, and having fire extinguishers on hand were strictly followed.

**Fig. 9:**
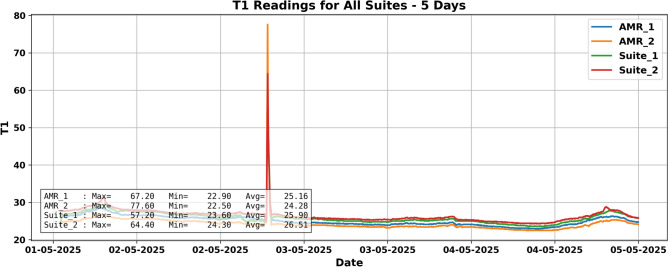
T1 readings across all sensor suites over 5 days. A sharp spike on May 4 indicates a simulated alcohol-based fire, with Suite2 recording the highest peak and AMRs detecting the rise earlier.

**Fig. 10:**
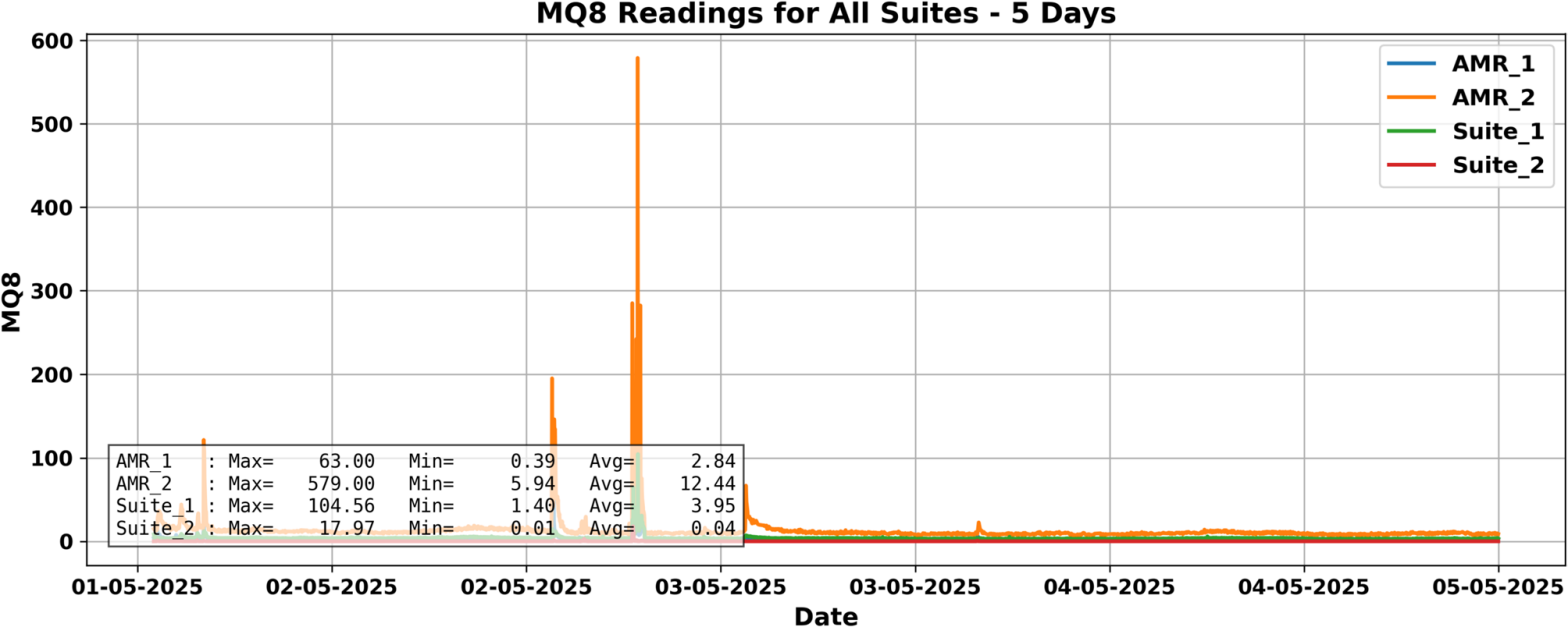
Hydrogen sensor readings during the same period. AMR2 shows a strong hydrogen peak ($\sim$758 ppm) on May 4, aligned with the fire event, confirming detection of combustion gases.

For instance, in a five-day test, a “fire event” on May 4, 2025, triggered notable sensor responses. T1 readings exceeded 43 °C in $\hbox {Suite}_2$, while AMRs registered earlier temperature rises due to mobility as shown in Fig. 9, Gas sensors MQ8 as shown in Fig. 10 and MQ4 detected hydrogen, validating spatial synchrony and sensor fusion potential.

Prior to synthetic generation, this real dataset served as the foundational baseline for the hybrid framework explained in the next section, informing both the statistical modeling of normal conditions and the temporal dynamics of hazard scenarios.

Basic descriptive statistics for the collected real dataset are provided in Supplementary Appendix E. Table E1 reports overall sensor statistics (count, mean, median, standard deviation, variance, min, max, range, skewness, kurtosis) computed across the combined 12-day normal baseline and five *Hazard_1* events as a real dataset sample, while Table E2 shows the same statistics computed separately for each sensor suite. These statistics were used to validate and parameterize the synthetic-augmentation methods so that generated signals reproduce the observed central tendency and dispersion without producing unrealistic extremes.

### Synthetic dataset generation

Due to the limited number of real hazard events and the inherent risks of reproducing them in physical industrial settings, the *RoboFusion* framework incorporates a dual-branch synthetic data generation pipeline. This extension aims to produce consistent synthetic signals, ensuring the availability of balanced, diverse training data suitable for evaluating AI-driven robotic hazard detection systems.

The synthetic pipeline consists of two parallel branches: (1) One for augmenting normal environmental conditions, (2) another for simulating realistic hazard progression patterns. These branches operate independently on the preprocessed real dataset to ensure isolation of statistical properties.

Each synthetic record is mapped onto the grid-based testbed environment, enabling it to be associated with specific spatial zones and AMR traversal paths. This design reflects real-world deployments in robotics, where sensing and environmental interaction are both location-aware and time-dependent.

#### Synthetic normal dataset generation

**Fig. 11:**
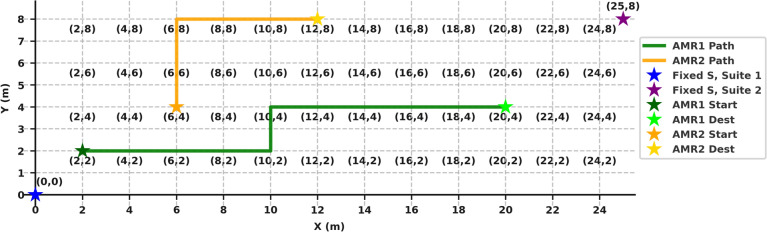
Testbed environment modeled as a grid with two fixed sensor suites and two AMRs.

The industrial testbed is divided into a virtual grid of 1$\times$1 m zones as illustrated in Fig. 11. AMRs were programmed to systematically traverse this layout using a relaxed Dijkstra algorithm, allowing efficient coverage of all spatial cells without excessive redundancy.

Fixed sensor suites are located at coordinates (0, 0) and (25, 8) as strategic locations within the testbed. They are positioned at the entrance and exit of the testbed to capture both incoming and outgoing ambient conditions. These areas coincide with zones of frequent material-handling activity and the presence of heat-generating equipment, which together represent the most likely sources of thermal and gas hazards. Placing the suites at these points also maximizes sensing coverage across the monitored area by providing complementary views of the environment’s inflow and outflow dynamics. While this layout proved optimal for our testbed, other industrial environments may define “strategic” sensor locations differently, depending on their spatial layout, airflow behavior, and dominant hazard types.

To replicate realistic fluctuations observed during non-hazardous operations (Normal Condition), five statistical augmentation techniques were employed. Each technique was carefully parameterized and validated to ensure it preserved the core structure of the original data from all the 12 sensors while introducing controlled variability. These techniques are summarized in Table 3.

**Table 3:** Augmentation Techniques with Configurable Parameters Description.

**Technique**	**Parameter**	**When to Use**
Noise Injection	Noise Level (%)	Simulates low-frequency sensor drift or noise
Time Warping	Time Distortion (%)	Models timing irregularities caused by environmental variability
Feature Scaling	Scaling Factor (%)	Represents amplitude fluctuations from calibration or seasonal changes
Window Slicing	Window Size	Creates additional overlapping time windows for sequence expansion
Interpolation	Polynomial Degree	Smooths transition or fill short data gaps using curve fitting

Following augmentation, statistical similarity was quantitatively assessed using (1) Pearson correlation coefficient^69^ as shown in Supplementary Appendix D Equation D1, (2) Kullback–Leibler divergence^70^ as shown in Supplementary Appendix D Equation D2, and (3) Basic statistics (mean, standard deviation, range). These metrics ensured that the augmented data did not introduce synthetic artifacts that could bias downstream learning models.

Although Table 4 presents evaluation metrics for the MQ4 gas sensor as a representative example, it is important to note that all augmentation techniques are systematically applied across the entire sensor array comprising 12 sensor channels per suite. The user may select one of the five augmentation methods independently, depending on the nature of the learning task or the desired variation (e.g., temporal jitter, drift simulation, or partial data loss).

Alternatively, the techniques can be executed sequentially in a pipeline, referred here as “merged augmentation”, where each method enhances a distinct aspect of the signal. In our example, the merged approach following the sequence (Smart Noise Injection $\rightarrow$ Time Warping $\rightarrow$ Feature Scaling) consistently produced the broadest signal range and most realistic variability without compromising evaluation metrics. These findings establish it as a robust configuration for generating synthetic normal-condition data in mobile robotic sensing applications.

This branch of the pipeline enables scenario diversity across time and space, crucial for training robust robotic perception systems capable of operating under various nominal conditions.

**Table 4:** Evaluation metrics between original and augmented MQ4 data for AMR_.

**Method**	**Min**	**Max**	**Mean**	**STD**	**Range**	**Correlation**	**KL_Divergence**
Noise Injection	103.18	184.40	150.99	4.48	81.22	1.0	0.0
Window Slicing	103.19	163.38	151.06	5.26	60.19	0.7	18.1
Time Warping	103.19	184.36	150.99	4.48	81.17	1.0	0.0
Interpolation	103.19	184.36	150.99	4.48	81.17	1.0	0.0
Feature Scaling	98.03	175.14	143.44	4.26	77.11	1.0	0.0
Merged Augmentation	98.01	175.11	143.44	4.26	77.10	1.0	0.0

#### Synthetic hazard dataset generation

While normal environmental conditions can be reliably augmented using statistical methods, real hazard events remain scarce, inconsistent, and risky to reproduce on a scale. To overcome this, the *RoboFusion* framework incorporates a structured synthetic modeling process that replicates the temporal dynamics of real hazard events across multiple hazard types. This synthetic hazard generation process enables the safe creation of high-fidelity signals to support robust learning in autonomous robotic systems deployed in industrial environments.

Unlike traditional sensor datasets which either simulate hazards using simple noise profiles or exclude them entirely, *RoboFusion* uniquely models real hazard dynamics using signal segmentation, function fitting, and spatial propagation. To the best of our knowledge, this is the first dataset-generation framework that synthesizes environmental hazards in both the temporal and spatial domains for industrial mobile robotics.

The methodology is designed to be generalizable across the three major hazard types observed in the dataset. Each hazard type exhibits a unique temporal and spatial signature, typically dominated by a leading sensor - the one most responsive to that hazard among the 12 sensors in each suite. For fire and high temperature events (Hazard_1 and Hazard_2), the leading sensor is T1, which records sharp rises in ambient temperature. For gas leakage events (Hazard_3), the most sensitive sensor is selected dynamically from the MQ family (MQ4, MQ5, MQ7, MQ8, or MQ135), depending on the gas type and dispersion pattern. The segmentation, fitting, and modeling steps are applied using this leading sensor as the primary reference.

#### Segmentation

Each real hazard event is segmented into four temporal segments based on the pattern observed in the leading sensor:

- Ignition: Initial deviation from baseline
- Growth: Rapid escalation in signal amplitude
- Peak: Sustained high-intensity phase
- Decay: Gradual return to ambient baseline

**Fig. 12:**
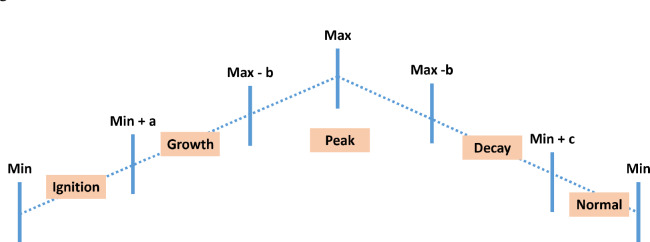
Temporal segmentation of a fire hazard event using the T1 temperature sensor.

Phase boundaries shown in Fig. 12 are identified using slope analysis of the leading sensor’s time series, leveraging the first derivative to detect inflection points (a, b, and c). Thresholds are determined empirically for each hazard type to ensure consistent segmentation.

#### Curve fitting

Each segment is modeled independently using a group of candidate regression functions including polynomial (up to degree 4), Gaussian, and exponential fits. Grid search is used to tune parameters for non-linear models. Candidate fitting functions are summarized in Supplementary Appendix D Table D1 for hazard_1 and T1 is the Leading Sensor in Suite_1. The best-fitting function per segment is selected based on two criteria: minimum root mean square error (RMSE) and maximum coefficient of determination ($R^2$).

**Fig. 13:**
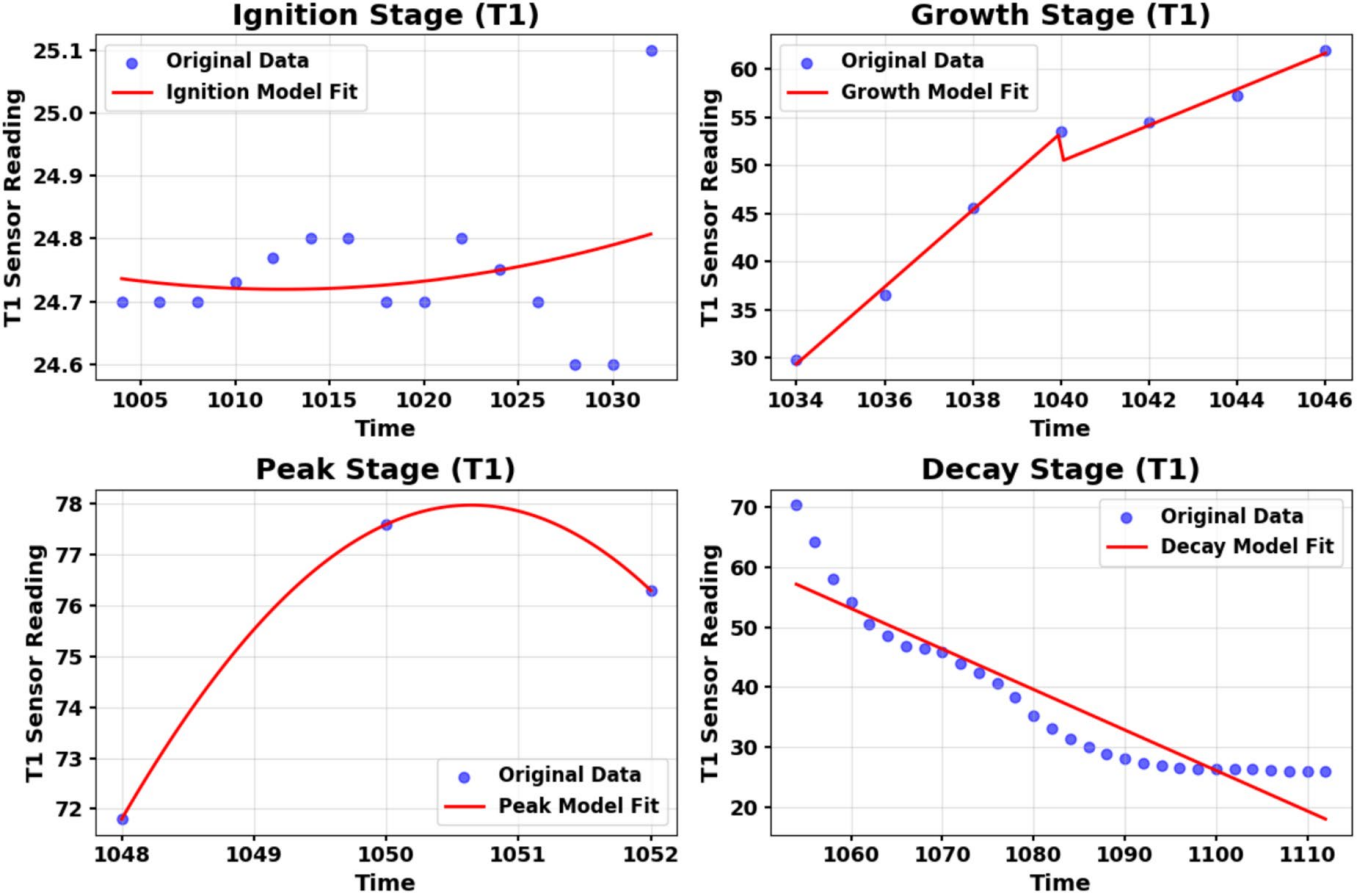
Fit models for T1 sensor with coefficients and evaluation metrics in hazard condition.

**Fig. 14:**
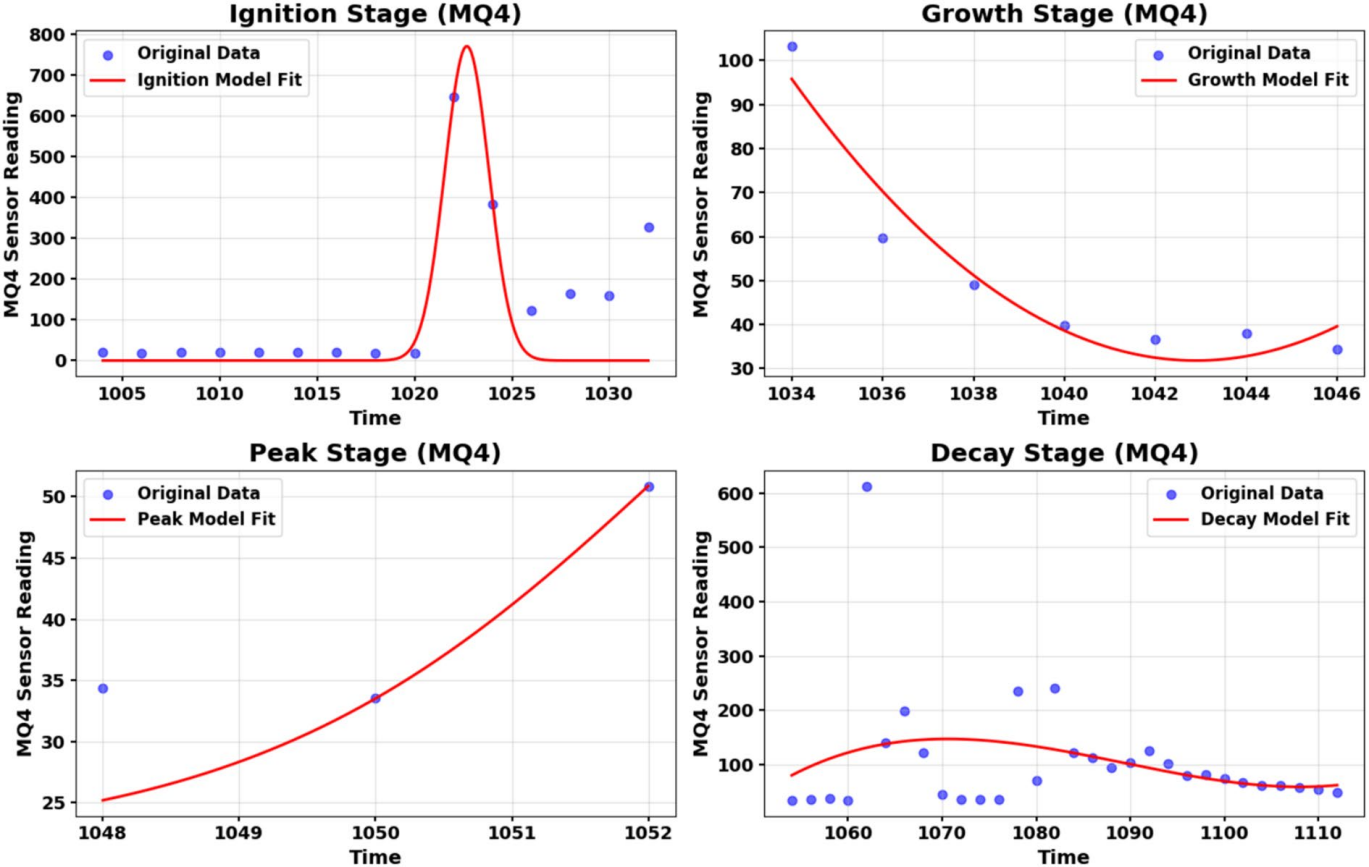
Fit models for MQ4 sensor with coefficients and evaluation metrics in hazard condition.

This process is repeated for all sensors within the four sensor suites, beginning with the leading sensor. An example of the fit models selected for T1 and MQ4 are shown in Figs. 13 and 14, respectively. The fitting coefficients for each model are stored per stage (ignition, growth, peak, decay) and used in the next synthesizing phase.

#### Composite hazard

Once the optimal models are identified for each of the four segments, they are combined to form a piecewise composite hazard function that represents each sensor in hazard condition. This function reconstructs the full synthetic signal in the time domain, as expressed in Supplementary Appendix D Equation D3.

#### Normalization process

To ensure signal realism and consistency with the real dataset’s scale, the composite synthetic signal is normalized to match the real amplitude ranges of the respective sensors. This scaling is achieved through linear normalization as shown in Supplementary Appendix D Equation D4.

#### Spatial scaling (Propagation modeling)

In real hazard scenarios, signal strength naturally decreases with increasing distance from the hazard origin. To replicate this behavior, the synthetic hazard signal is adjusted using a logistic attenuation function applied over the Euclidean distance to the source zone. This spatial modulation ensures that AMRs perceive hazard intensity in a realistic manner that depends on their proximity and traversal path. The resulting spatially varying intensity is computed using Supplementary Appendix D Equation D5^71^.

The temporal evolution of the hazard intensity follows a logistic growth model, as defined in Supplementary Appendix D Equation D6^72^. The parameters of this model are tuned using Bayesian optimization to produce realistic propagation curves that match the desired hazard behavior, whether fast, gradual, or delayed.

By combining segmented curve fitting, amplitude normalization, and spatial scaling, the synthetic hazard generation process provides flexible control over hazard dynamics. From a single real hazard event, the framework can generate limitless variations of the same hazard with different speeds, shapes, and intensities. These outcomes are governed by tunable parameters such as segment curvature, normalization range, and attenuation slope. This parameterized control enables the simulation of realistic and diverse hazard behaviors, directly contributing to improved generalization of ML models by exposing them to a broader distribution of hazard manifestations during training.

The final step involves injecting the modeled hazard signals into the dense synthetic normal dataset generated earlier. Each injection occurs at a randomly selected timestamp and zone location to simulate diverse scenarios. Multiple synthetic hazards can be injected into a single sequence to model temporal overlaps or spatially distributed events across space.

This method enables the same hazard event to be rendered at different zones, durations, and intensities, creating diverse training scenarios for autonomous mobile robots.

A representative row from the generated synthetic dataset is shown in Table 5. It maintains the same schema and labeling strategy as the real dataset, including suite ID, timestamp, spatial location, and all 15 sensor readings.

**Table 5:** Synthetic normal dataset schema.

Date	Time	Suite_ID	Location	T1	T2	H%	P	AL	Dust	MQ2	MQ4	MQ5	MQ7	MQ8	MQ135	Condition
Schema (column names) only; values omitted for brevity.																

### Summary

*RoboFusion* generates synthetic environmental datasets for hazard prediction by combining real sensor measurements from AMRs and fixed sensor nodes deployed across a mapped facility. The collected data are first labeled as normal or hazardous, and each hazard event is decomposed into four universal phases, namely Ignition, Growth, Peak, and Decay, which capture the typical temporal evolution of physical hazards. For every hazard type, the most sensitive (leading) sensor is identified and used to model how the event unfolds. Synthetic data are then produced through two pipelines: one that augments normal condition data to create natural variability, and another that models hazard phases using fitted temporal curves and spatial propagation functions. This structure enables *RoboFusion* to generate realistic and controllable datasets for training hazard prediction models.

## Results

This section presents a comprehensive validation of the *RoboFusion* framework using a range of ML models applied to both real and synthetic datasets. The objective is to evaluate how effectively these models can detect hazardous environmental conditions under varying data availability scenarios and training constraints. To ensure broad coverage, four classifiers were selected, namely Random Forest (RF), Support Vector Machine (SVM with RBF kernel), Extreme Gradient Boosting (XGBoost), and a shallow Multi Layer Perceptron (MLP), which together represent tree based, kernel based, ensemble, and neural architectures. All models were trained and evaluated as binary classifiers (*Normal*, *Hazard*) using precision, recall, and F1 score as the primary performance metrics.

Three main data-domain scenarios were examined (S1, S2, and S3), summarized in Table 6. Each scenario differs in the type of data used for training and testing, and collectively they evaluate baseline real data, synthetic-only, and cross-domain performance.

**Table 6:** Overview of the three main data-domain scenarios used for model evaluation, showing training–testing data types and corresponding result tables.

**Scenario ID**	**Training data**	**Testing data**	**Purpose**	**Results table**
S1	Real dataset	Real dataset	Evaluate baseline model performance using only real data	Table 9
S2	Synthetic dataset	Synthetic dataset	Assess consistency and learning behavior using purely synthetic data	Table 10
S3	Synthetic dataset	Real dataset	Evaluate transferability of synthetic-trained models to real hazards	Table 11

### Real dataset evaluation (S1)

A real-world dataset was collected over 180 days, comprising $\sim$1,036,800 samples from the four sensor suites. Fifteen hazard events were recorded and labeled, covering three distinct hazard types: Hazard_1 $\rightarrow$ Fire (5 events), Hazard_2 $\rightarrow$ High temperature fluctuations (4 events), Hazard_3 $\rightarrow$ Gas leakage (6 events). Each hazard spanned 30 to 120 minutes, enabling robust yet limited supervised training.

Table 7 summarizes the number of samples collected during the 180-day deployment, categorized into normal data and three hazard classes. As shown, normal conditions account for over 99.6% of all samples, while hazard events collectively represent less than 0.5%. This extreme imbalance reflects the natural scarcity of real hazards in controlled industrial settings and underscores the importance of synthetic augmentation to ensure adequate representation for supervised learning models.

**Table 7:** Summary of collected samples across normal and hazard conditions during the 180-day deployment.

**Data type**	**Duration/no. of events**	**No. of samples**	**Percentage (%)**
Normal Data	180 Days	1,036,800	99.61%
Hazard_1	5 Events	1,684	0.16%
Hazard_2	4 Events	1,032	0.10%
Hazard_3	6 Events	1,348	0.13%
**Total**	–	**1,040,864**	**100%**

Within Scenario **S1**, four test cases (**T1** to **T4**) were designed to explore how different real data configurations, such as temporal splits, reduced hazard density, and unseen hazard events, affect classifier performance. These test cases, summarized in Table 8, correspond to the first row presented in Scenario **S1** in Table 6.

**Table 8:** Definition of the four test cases (T1–T4) under Scenario S1 (Real $\rightarrow$ Real).

**Test case**	**Description**	**Classifier**	**Training**	**Testing**	**Purpose**
T1	Baseline (70%–30% temporal split on full dataset)	RF	180 days	180 days (reuse)	Establish baseline performance on real data.
T2	Leave-One-Hazard-Out (LOHO) validation	RF	180 days	45 days (reuse)	Assess generalization to unseen hazard events.
T3	Reduced-data experiment	RF	50 days	12 days (non-overlapping)	Evaluate model performance with limited real-data availability.
T4	Reduced-data experiment	XGBoost	50 days	12 days (non-overlapping)	Compare classifier robustness under the same reduced-data condition.

Table 8 summarizes the four test configurations (T1–T4) designed under Scenario S1 (Real $\rightarrow$ Real) to examine the influence of data partitioning, hazard exposure, and data availability on model robustness. The baseline test case (T1) adopts a conventional 70–30% temporal split to establish reference performance on the full real dataset. The Leave-One-Hazard-Out validation (T2) evaluates model generalization to unseen hazard events. Reduced-data experiments (T3 and T4) simulate constrained data conditions using shorter, non-overlapping periods, with T3 employing Random Forest and T4 using XGBoost to compare algorithmic resilience under identical data scarcity. Together, these configurations systematically test temporal, structural, and model-level generalization within purely real-world data scenarios.

**Table 9:** Hazard classification performance under real-world constraints.

**Test case ID**	**Hazard_1 F1**	**Hazard_2 F1**	**Hazard_3 F1**	**Macro F1**	**Conclusion**
T1	0.98	0.98	0.98	0.99	Perfect scores; overfitting.
T2	0.00	0.00	0.00	0.25	Total failure of unseen hazards.
T3	0.60	0.94	0.06	0.65	Improved generalization.
T4	0.39	0.43	0.10	0.48	XGBoost underperforms RF.

The results shown in Table 9 indicate that even with 180 days of real-world data, generalization fails unless hazards are duplicated or densely represented. The most striking failure is in T2, where the RF model trained on all available hazards except one instance (LOHO) per hazard type completely failed to detect the unseen events (F1 = 0.00 for Hazard_1 and Hazard_2). This highlights a critical gap: real data alone is not sufficiently diverse to support ML model generalization to new but semantically similar hazards.

To understand why real-world hazard classification underperforms, we analyzed intra-class sensor variability within each hazard class across all 15 hazards events. For each event, we calculated the mean and standard deviation of all sensor readings:

1. **Hazard_1** showed moderate consistency across gas and thermal sensors, although signal magnitudes varied between locations.
2. **Hazard_2** exhibited broader fluctuations, particularly in MQ135 and MQ2, indicating sensitivity to local airflow and ambient dynamics.
3. **Hazard_3** was the most inconsistent, with wide differences in response shape and peak amplitude caused by variations in dispersion conditions and source proximity.

**Fig. 15:**
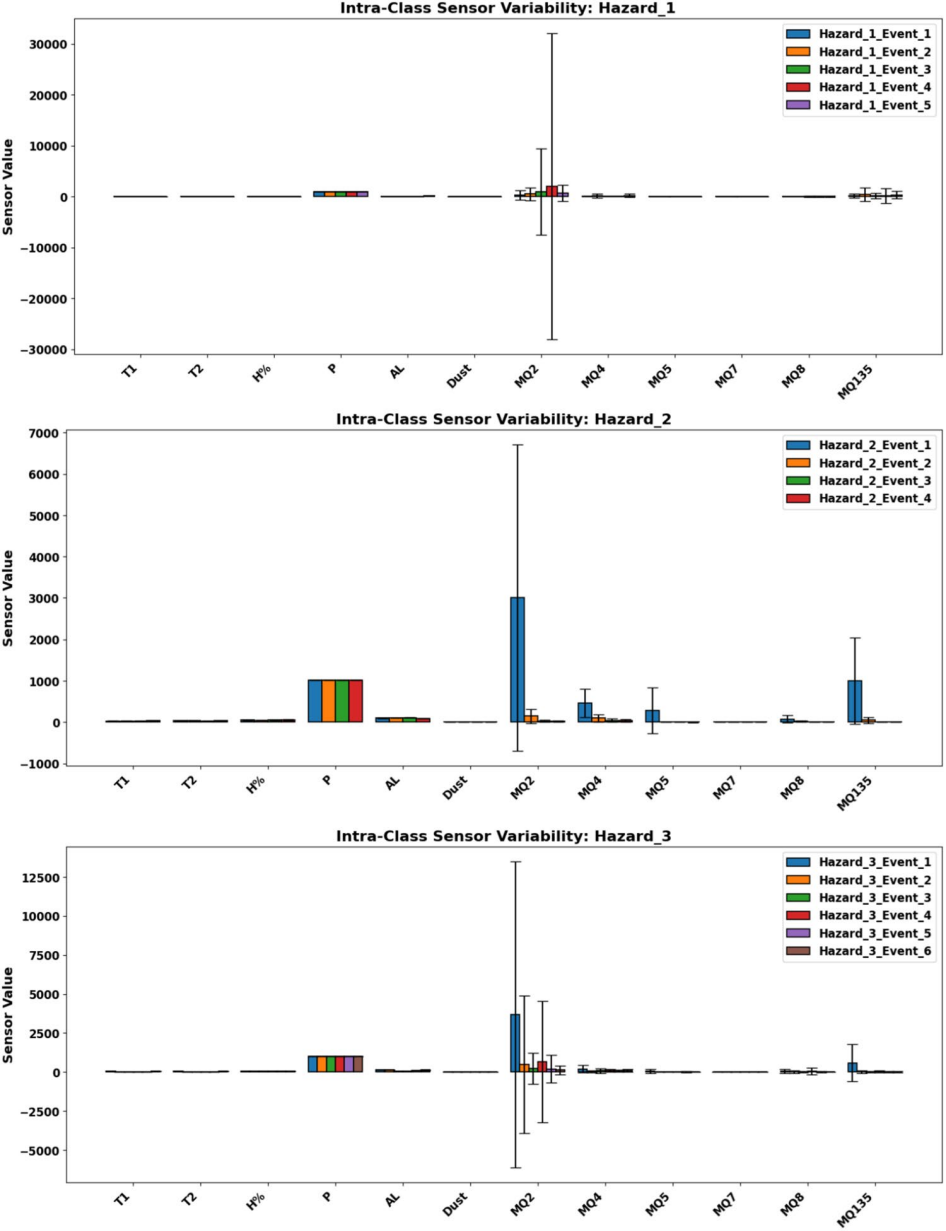
Intra-class variability across hazard types — Mean ± standard deviation.

This intra-class variability prevents the formation of stable sensor space patterns; without consistent features across events of the same hazard type, supervised models struggle to learn reliable decision boundaries. This explains the observed class wise differences in model detectability, especially the reduced accuracy for gas leak events, and underscores the importance of synthetic data in enhancing per class consistency.

These results, visually illustrated in Fig. 15, confirm that limited and internally diverse real hazard data provide weak support for generalizable ML, thereby highlighting the value of the hybrid synthetic-augmentation strategy proposed in *RoboFusion*.

### Synthetic dataset evaluation (S2)

To address the scarcity and inconsistency of real hazards, the *RoboFusion* framework generated a synthetic dataset containing 360 days of normal operations and 100 staged hazard sequences with varied onset, magnitude, and duration (These synthetic data sizes can be extended to limitless sizes). Models trained and tested on this synthetic dataset showed consistently high performance as shown in Table 10.

**Table 10:** Synthetic dataset evaluation (Train on Synthetic $\rightarrow$ Test on Synthetic).

**Model**	**Accuracy**	**Normal precision**	**Hazard precision**	**Normal recall**	**Hazard recall**	**Normal F1**	**Hazard F1**
RF	0.98	0.98	0.97	0.99	0.99	0.98	0.98
SVM (RBF)	0.96	0.96	0.95	0.97	0.97	0.96	0.96
XGBoost	0.97	0.97	0.96	0.98	0.98	0.97	0.97
MLP	0.95	0.95	0.94	0.95	0.95	0.95	0.94

### Cross-domain validation (S3)

To test generalization, we trained models only on synthetic data and evaluated them on real hazard events. Despite the domain shift, synthetic-trained models substantially outperformed real-only models in recall and F1 score for all hazard types as shown in Table 11.

**Table 11:** Cross-domain validation (Train on Synthetic $\rightarrow$ Test on Real).

**Model**	**Accuracy**	**Normal precision**	**Hazard precision**	**Normal recall**	**Hazard recall**	**Normal F1**	**Hazard F1**
RF	0.92	0.93	0.83	0.91	0.87	0.92	0.85
SVM (RBF)	0.90	0.92	0.75	0.89	0.84	0.91	0.79
XGBoost	0.91	0.92	0.81	0.90	0.86	0.91	0.83
MLP	0.85	0.87	0.69	0.84	0.78	0.85	0.73

**Fig. 16:**
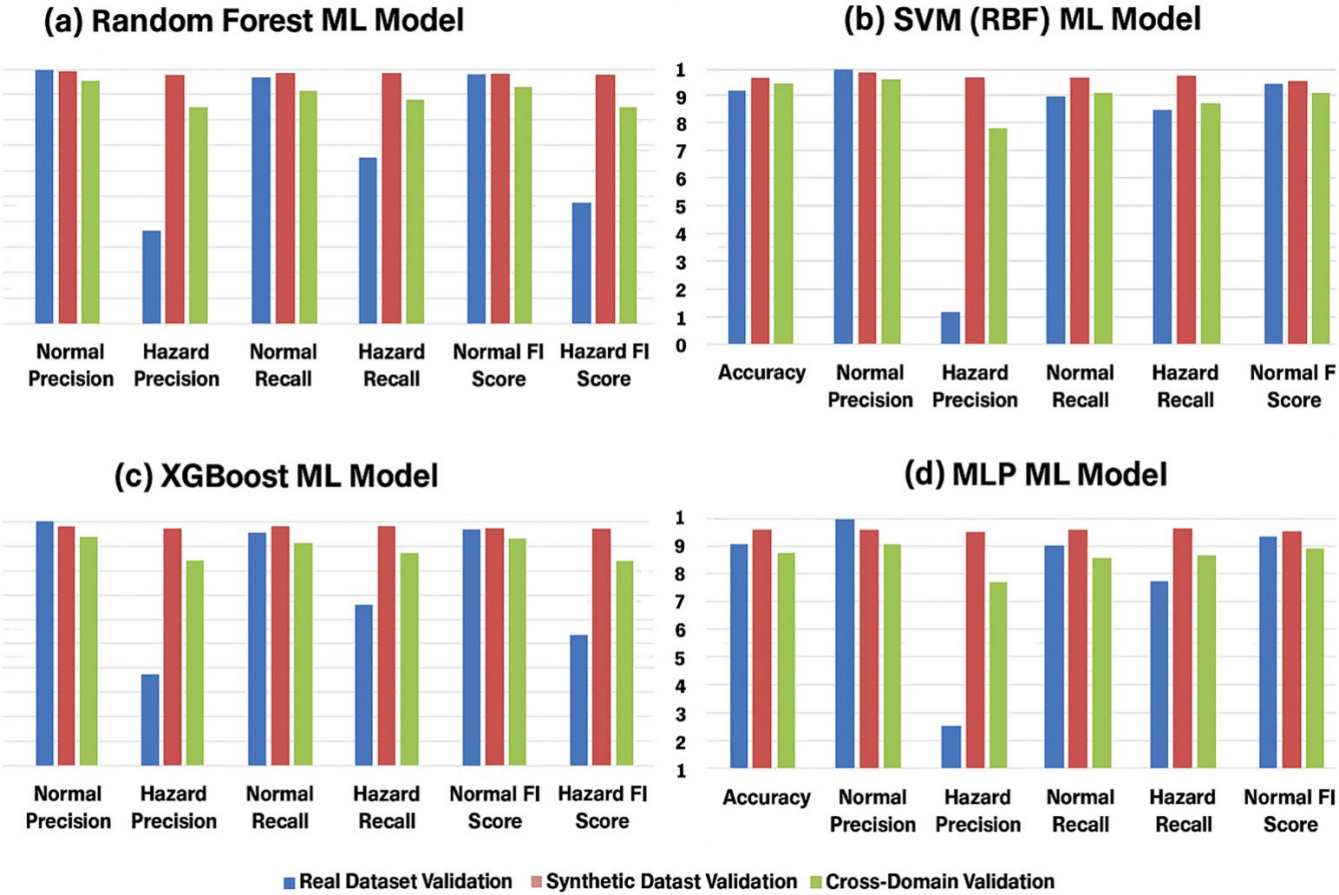
Model performance across real, synthetic, and cross-domain validation scenarios. (**a**) RF; (**b**) SVM (RBF); (**c**) XGBoost; (**d**) MLP.

This performance recovery demonstrates that synthetic hazards, when modeled across diverse parameters, help models generalize even to unseen real world events. Figure 16 compares the performance of the four machine learning models across real, synthetic, and cross domain validation scenarios. Each subplot illustrates key classification metrics versus each ML model.

To further examine the statistical robustness of the reported results, a 5-fold stratified cross-validation (CV) procedure was employed following standard machine-learning evaluation practices.

In this protocol, the dataset is randomly partitioned into five equally sized subsets (folds) while preserving the proportion of normal and hazard samples within each split. During each iteration, four folds are used for training and one for testing; this process repeats five times such that each sample appears exactly once in a test fold. The reported values correspond to the mean ± standard deviation (SD) of precision, recall, and F1-score across folds. This approach quantifies the stability and reproducibility of model performance under resampling, ensuring that the reported metrics are not artifacts of a single train–test configuration.

To complement the single-run experiments reported above and address the reviewer’s concern regarding statistical validation, three validation scenarios were examined:

1. **Real** $\rightarrow$ **Real (5-fold CV):** The real dataset was evaluated under stratified 5-fold cross-validation. The low standard deviations ($\sigma \approx 0.001$–0.005) indicate consistent classifier behavior across folds despite class imbalance.
2. **Synthetic** $\rightarrow$ **Synthetic (5-fold CV):** The synthetic dataset was validated using the same CV protocol to confirm internal coherence. Both Random Forest and XGBoost achieved near-perfect separability (F1 $\approx$ 0.99, $\sigma < 0.003$), confirming the dataset’s structural consistency.
3. **Synthetic** $\rightarrow$ **Real (single cross-domain evaluation):** Models trained on the full synthetic dataset were tested once on the complete real dataset to measure domain transferability. This setup intentionally avoids cross-validation to maintain domain independence.

These results align with those of the original cross-domain experiment (Table 11), confirming that the synthetic data preserve real-world statistical characteristics. The consolidated outcomes are summarized in Table 12, combining intra-domain and cross-domain validations in a unified format.

**Table 12:** Consolidated cross-validation and cross-domain results (Scenarios 1–3).

Scenario/Model	Precision (mean ± SD)	Recall (mean ± SD)	F1-score (mean ± SD)
**S1 – Real **$\rightarrow$**Real (5-fold CV)**			
Random Forest	0.501 ± 0.002	0.518 ± 0.003	0.362 ± 0.005
XGBoost	0.500 ± 0.001	0.491 ± 0.002	0.300 ± 0.004
**S2 – Synthetic**$\rightarrow$**Synthetic (5-fold CV)**			
Random Forest	0.987 ± 0.002	0.986 ± 0.003	0.987 ± 0.002
XGBoost	0.991 ± 0.001	0.992 ± 0.001	0.992 ± 0.001
**S3 – Synthetic **$\rightarrow$**Real (single evaluation)**			
Random Forest	0.501	0.518	0.362
XGBoost	0.500	0.491	0.300
SVM (RBF)	0.494	0.387	0.292
MLP	0.486	0.253	0.286

Table 12 presents the consolidated results of the three validation scenarios. Scenarios 1 and 2 report 5-fold cross-validation outcomes (mean ± SD), whereas Scenario 3 represents a single cross-domain evaluation in which models trained on synthetic data are tested on real data. The low fold variance observed in Scenarios 1 and 2 ($\sigma \approx 0.001$–0.005) confirms the stability and reproducibility of model performance, while the consistency observed in Scenario 3 verifies the alignment between synthetic and real domains.

These validation experiments demonstrate that the *RoboFusion* framework produces reproducible results under resampling and maintains stable performance when transferring from synthetic to real environments, thereby reinforcing the statistical credibility of the reported findings.

### Quantitative similarity between real and synthetic signals

To quantitatively assess how closely the synthetic dataset reproduces the temporal dynamics of the real sensor data, three complementary similarity metrics were applied between each real–synthetic signal pair: Dynamic Time Warping (DTW), Fréchet distance, and Energy distance. Each metric captures a distinct aspect of realism:

- **DTW** measures temporal alignment between two sequences that may differ in timing or duration, identifying how well the synthetic signal follows the time-evolving shape of the real signal.
- **Fréchet distance** quantifies the overall geometric similarity between curves, being sensitive to both amplitude and shape.
- **Energy distance** compares statistical distributions, indicating whether the synthetic signals share the same overall value distribution as the real data.

**Fig. 17:**
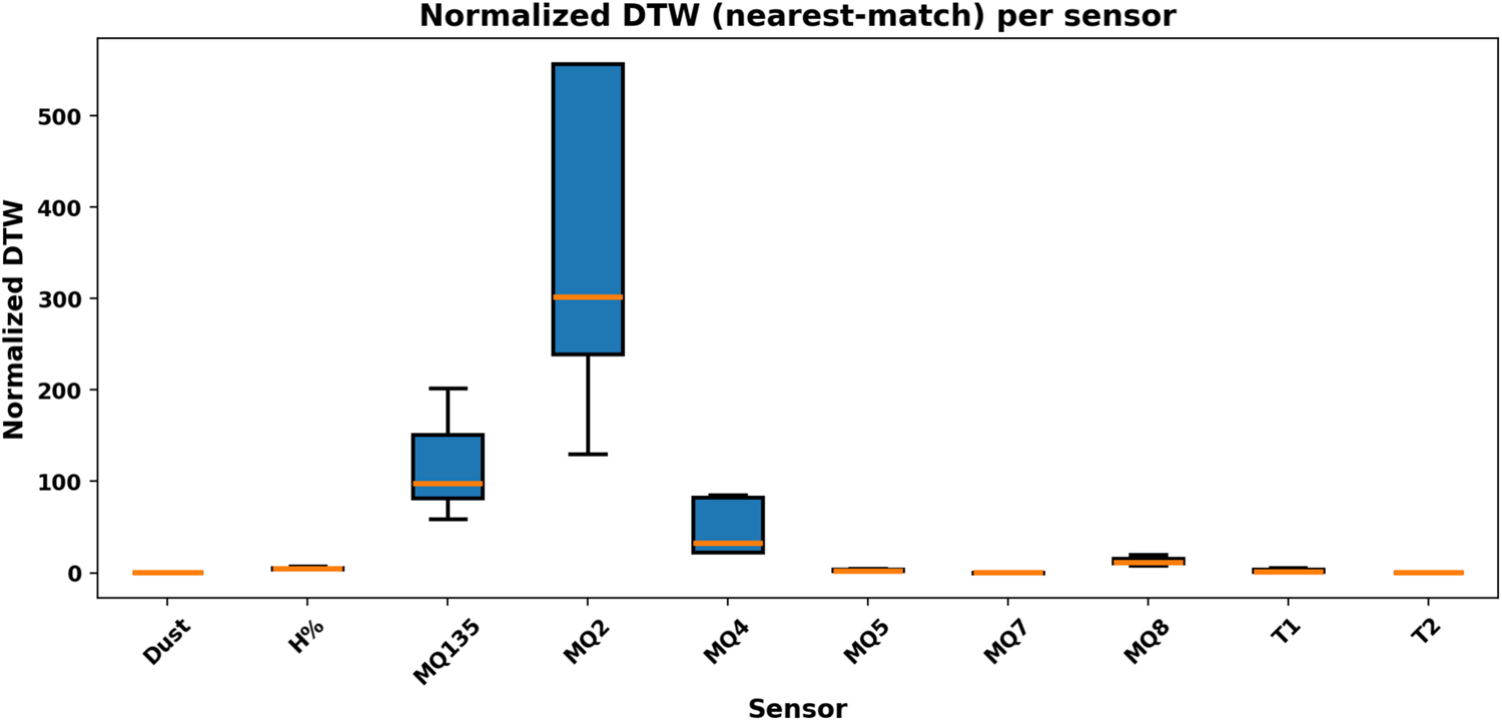
Normalized Dynamic Time Warping (DTW) similarity between real and synthetic signals across all sensors.

The analysis was performed for all twelve sensors using five real *Hazard*_1_ (fire) events and twelve synthetic counterparts generated by the *RoboFusion* framework. Results are summarized in Table 13 and visualized in Fig. 17.

Sensors primarily responsive to thermal and particulate phenomena (T1, T2, Dust, H%, P, AL) show very low normalized DTW values ($\approx$ 0.8–2.5) and Energy Distances < 1.6, confirming that the synthetic generator effectively captures the real fire dynamics. Gas sensors (MQ2, MQ4, MQ135) exhibit higher distances because they play a minor role in fire events and remain close to baseline; thus, their apparent mismatch is physically expected. These sensors become dominant in *Hazard*_3_ (Gas Leak) scenarios.

**Table 13:** Quantitative similarity metrics (DTW, Fréchet, and Energy distances) between real and synthetic *Hazard*_1_ signals across all sensors.

**Sensor**	**DTW (Mean)**	**Normalized DTW**	**Fréchet distance**	**Energy distance**
T1	435.8	0.88	7.34	1.11
T2	497.3	0.91	9.27	1.42
H%	2231.4	5.00	35.01	1.30
P	912.6	2.04	15.52	1.25
AL	1201.9	2.45	16.09	1.46
Dust	510.3	0.94	6.82	1.64
MQ2	223216.7	512.90	117150.83	5.90
MQ4	20055.4	49.36	1424.78	6.12
MQ5	18243.8	41.02	1210.67	5.35
MQ7	6841.2	14.23	372.55	3.48
MQ8	9324.4	19.57	587.41	3.91
MQ135	52529.4	118.52	7634.26	4.68

## Discussion

The evaluation of the *RoboFusion* framework across real, synthetic, and cross domain validation scenarios reveals critical insights into the limitations of real world datasets for hazard detection and the transformative potential of synthetic datasets.

The 180 day real dataset, which included fifteen controlled hazard events, provided a valuable testbed for exploring model performance under realistic operational conditions. However, even when models were trained on nearly all available real hazard data, with just one event withheld for each hazard type, the results showed complete failure to generalize. Specifically, in the LOHO scenario, F1 scores dropped to zero for both fire and high temperature hazards, which exposed a significant weakness in relying solely on real data. These findings confirm that hazard diversity in real datasets is simply too limited to support robust machine learning generalization.

Further analysis of intra-class sensor behavior revealed substantial variability in how different hazard instances express themselves across sensing modalities. In some cases, the same hazard type led to different sensor peaks, durations, or rates of change depending on spatial location, environmental dynamics, or sensor position. This inconsistency prevents models from learning stable, repeatable decision boundaries. Even increasing the density of hazard events only partially recovered performance, and often for a subset of hazard classes.

By contrast, the synthetic dataset generated by *RoboFusion* offers consistent and structured hazard representations while maintaining statistical variability. Synthetic hazards are constructed using stage-wise curve fitting, distance-based spatial modeling, and tunable intensity dynamics. These design elements enable the generation of hundreds of hazard profiles with controlled diversity, creating training conditions that support generalized learning.

Cross domain experiments further underscore this point. Models trained exclusively on synthetic data achieved hazard F1 scores up to 0.85 when tested on real world events, far outperforming models trained only on real data. This performance gap reinforces the idea that the bottleneck in hazard detection is not the classifier architecture, but the scarcity and inconsistency of labeled hazard data.

In safety-critical environments, where real hazards are rare, dangerous, and often non-reproducible, the *RoboFusion* framework enables scalable and transferable AI-based hazard detection. Rather than replacing real data, synthetic augmentation fills in the statistical and semantic gaps that real datasets cannot cover. Future work will extend the hazard types modeled, explore domain adaptation techniques, and validate performance across varied industrial layouts to further enhance the robustness and adaptability of the framework.

While *RoboFusion* successfully integrates real and synthetic data to reproduce realistic environmental dynamics, some of the current validation was limited to the Hazard_1 (fire) event class and a single-site deployment. Future work will extend the framework to encompass additional hazard types such as high temperature fluctuations (Hazard_2) and gas leaks (Hazard_3), incorporate domain-adaptation metrics (e.g., MMD and t-SNE) for cross-domain evaluation, and validate the system across multiple industrial layouts to enhance robustness and generalization.

Unlike purely data-driven generators such as SMOTE and TimeGAN, which focus on statistical or latent-space interpolation, *RoboFusion* blends empirical signal dynamics with analytically modeled hazard evolution. This hybrid strategy preserves physical interpretability while maintaining statistical diversity, avoiding mode collapse and unrealistic correlations often observed in fully generative methods.

## Conclusions

This paper introduced RoboFusion, an integrated robotic framework that enables real-time environmental sensing and synthetic hazard modeling within industrial environments. By combining AMRs with fixed sensor suites, NFC-based localization, and embedded edge computing, *RoboFusion* offers a practical and low-cost solution for mobile robotic monitoring in structured facilities.

A key innovation of this work is the hybrid dataset generation pipeline, which merges real telemetry from mobile and static suites with synthetic hazard simulations. The synthetic generation strategy models realistic multi-phase hazard dynamics and spatial propagation, offering a scalable and reproducible method to expose robotic systems to rare safety-critical events without physical risk.

Experimental results demonstrated that machine-learning models trained on synthetic data significantly outperform models trained on real data alone, particularly in detecting underrepresented hazard events. In cross-domain validation, synthetic-trained models preserved generalization, with hazard F1-scores reaching 0.85 (Random Forest) and 0.79 (SVM). This highlights the utility of synthetic datasets for pre-training or augmenting robotic perception systems.

As a whole, *RoboFusion* serves as both a robotic platform and a data generation tool for advancing intelligent sensing in hazardous environments. Its modular architecture, open-access dataset, and deployable in real-world industrial conditions make it valuable for researchers in robotics, AI, and smart manufacturing.

Future work will focus on integrating *RoboFusion* with adaptive robot control policies, online learning, and transfer learning across different industrial environments. We also plan to explore domain adaptation techniques that allow models trained on synthetic hazards to adapt quickly to new facilities, even when limited real data is available. Expanding real-world hazard recordings and applying the framework to additional robotic platforms will help validate *RoboFusion*’s generalizability and impact across a wider range of conditions.

## Supplementary Information

Supplementary Information.

## Data Availability

The real and synthetic datasets generated and analysed during the current study are publicly available in the GitHub repository at: https://github.com/Amr-Khamis-84/RoboFusion-Dataset.
