# Supplementary material for: Hybrid real-synthetic dataset framework for robotic hazard detection in industrial environments
Source: Sci Rep. 2026 Jan 12;16:1628. doi: 10.1038/s41598-025-33603-5 (PMC12800294; doi:10.1038/s41598-025-33603-5)
Supplement: Supplementary file 1 — Supplementary Information. [file 41598_2025_33603_MOESM1_ESM.pdf]

Appendix A: Acronyms List

Table A1: List of acronyms and their expanded definitions used throughout the *RoboFusion* framework.

Acronym	Expanded Definition	Acronym	Expanded Definition
AASTMT	Arab Academy for Science, Technology and Maritime Transport	AI	Artificial Intelligence
AGV	Automated Guided Vehicle	AL	Altitude
AMR	Autonomous Mobile Robot	ANN	Artificial Neural Network
API	Application Programming Interface	CNN	Convolutional Neural Network
CPU	Central Processing Unit	CSV	Comma-Separated Values
DL	Deep Learning	DTW	Dynamic Time Warping
GNN	Graph Neural Network	GPS	Global Positioning System
IMU	Inertial Measurement Unit	IoT	Internet of Things
LiDAR	Light Detection and Ranging	MCU	Microcontroller Unit
ML	Machine Learning	MQ	Metal Oxide (Gas Sensor Series)
PCB	Printed Circuit Board	RAM	Random Access Memory
RNN	Recurrent Neural Network	SD	Standard Deviation
SNR	Signal-to-Noise Ratio	ToF	Time of Flight
USB	Universal Serial Bus	Wi-Fi	Wireless Fidelity
AMR_1	Autonomous Mobile Robot 1	AMR_2	Autonomous Mobile Robot 2
ALU	Arithmetic Logic Unit	ANOVA	Analysis of Variance
AQL	Air Quality Level	AQI	Air Quality Index
BMS	Building Management System	BLE	Bluetooth Low Energy
CFD	Computational Fluid Dynamics	CSV	Comma Separated Values
DHT	Digital Humidity and Temperature sensor	DOF	Degree of Freedom
DLPS	Device Level Power Supply	DRL	Deep Reinforcement Learning
DSP	Digital Signal Processor	EEPROM	Electrically Erasable Programmable Read-Only Memory
ESP32	Espressif ESP32 microcontroller	FOV	Field Of View
FTP	File Transfer Protocol	GEAF	Gas Exchange Assist Fan
GUI	Graphical User Interface	HDD	Hard Disk Drive
Hz	Hertz	IAQ	Indoor Air Quality
I/O	Input/Output	IoRT	Internet of Robotic Things
I^2C	Inter-Integrated Circuit	ToF	Time-of-Flight
IoT	Internet of Things	VDC	Volts Direct Current
I^2C	Inter-Integrated Circuit	VOC / VOCs	Volatile Organic Compounds
IP	Internet Protocol	Wi-Fi	Wireless Fidelity
ISC	Industry Service Complex	WPA2-PSK	Wi-Fi Protected Access 2 – Pre-Shared Key
JSON	JavaScript Object Notation	XGBoost	Extreme Gradient Boosting
Li-ion	Lithium-ion		

Appendix B: Dataset Benchmarking and Coverage Analysis

Table B1: Summary of environmental datasets — Bibliography

Ref	Key Feature	Application Domain	Geographical Scope
[35]	Window behavior prediction in offices	Building Behavior Modeling	Germany
[36]	Thermal soil simulation in urban canyons	Urban Climate	France
[37]	Linking IEQ to comfort and cognition	IEQ & Comfort	UAE
[38]	IEQ and cognitive function impact	IEQ & Health	USA
[39]	IEQ comparison: green vs. conventional buildings	IEQ Comparison	China
[40]	Review: ML for energy profiling	Urban Energy Modeling	N/A
[41]	Adaptive comfort modeling	Thermal Comfort	SE Asia
[42]	IEQ & occupant satisfaction model	IEQ & Satisfaction	China
[43]	Modular building simulation engine	HVAC Simulation	Global
[44]	Simulation: airflow, daylighting, HVAC	Building Simulation	Global
[45]	CHTC model for windward facades	Building Aerodynamics	Global
[46]	Health risk from indoor PM2.5	Environmental Health Risk	China
[47]	Long-term IAQ in homes with ventilation	Residential IAQ	China
[48]	IAQ study in Brazilian universities	University IAQ	Brazil
[49]	Nationwide IAQ in schools	School IAQ	China
[50]	IoT-based IAQ in a "living lab"	Smart Home IAQ	South Korea
[51]	Controlled vs. uncontrolled climate data	Climate Monitoring	Colombia
[52]	ETS pollutant inhalation modeling	Pollution Risk	USA
[53]	CO_2-based IAQ in cold climates	School Comfort	China
[54]	Evaluation of indoor living walls	Green Design	UK
[55]	Low-cost IoT air quality monitoring	Low-Cost Sensing	India
[56]	Neural calibration for benzene sensing	Gas Sensor Calibration	Italy
[57]	Post-occupancy IEQ evaluation	Occupant Feedback	USA
[58]	IoT + ML for industrial AQ	Industrial Air Quality	Spain, Colombia
[59]	Comfort & energy multi-agent control	Building Automation	Malaysia
[60]	CO_2 emissions vs. building height	Design & Emissions	South Korea
[61]	Saudi Arabia indoor dataset	Regional Smart Home	Saudi Arabia
RoboFusion	Hybrid real–synthetic dataset using AMRs for hazard detection and environmental awareness	Industrial Facility	Egypt (Alexandria)

Table B2: Summary of environmental datasets — Technical Review

Ref	Sensor Types Used	System Mobility	Data Freq.	Duration	Dataset	ML
[35]	Indoor temp, surface temp, CO_2, wind, solar radiation, rain, illuminance, occupancy, window status	Fixed	10 min	2 years	✓	✓
[36]	None (simulation tool)	Fixed	N/A	N/A	X	X
[37]	Air temp, radiant temp, humidity, illuminance, air speed, noise, outdoor temp	Fixed	15 min	7 days	X	X
[38]	CO_2, temp, humidity, sound, VOCs, NO_2, O_3, PM2.5, pressure, light	Fixed	1 min	6 days	X	X
[39]	Temp, humidity, CO_2, illumination, noise, PM2.5	Fixed	5 min	1 year	X	X
[40]	Smart meters, BMS data (aggregated)	Fixed	N/A	N/A	X	✓
[41]	Temp, RH, air speed, MRT (ASHRAE RP–884)	Fixed	5 min	1 year	X	X
[42]	Temp, RH, air velocity, CO_2, light, noise	Fixed	1 min	2 years	X	X
[43]	None (simulation tool)	Fixed	User-def.	User-def.	X	X
[44]	None (simulation tool)	Fixed	User-def.	User-def.	X	X
[45]	None (CFD only)	Fixed	User-def.	N/A	X	X
[46]	PM2.5 (heavy metals via filter/lab assays)	Fixed	24 h	2 months	X	X
[47]	PM2.5, CO_2, temp, humidity, VOCs, HCHO	Fixed	60 min	1 year	X	X
[48]	CO_2, PM10, temp, humidity	Fixed	5 min	1 week	X	X
[49]	PM2.5, CO_2, TVOC, temp, humidity	Fixed	5/50 min	9 months	X	X
[50]	Temp (DHT22), RH, PM1/2.5/10 (PMS7003), CO_2 (T6713)	Fixed	1 min	1 month	X	X
[51]	Xiaomi Mijia BLE (Temp, RH)	Fixed	2 min	2 months	✓	X
[52]	None (literature-based emission estimates)	Fixed	User-def.	User-def.	X	X
[53]	CO_2, temp, humidity	Fixed	5 min	5 days	X	X
[54]	Temp, RH, light, air velocity	Fixed	N/A	N/A	X	X
[55]	MQ135, MQ7	Portable	15 s	N/A	X	✓
[56]	Multi-gas array, temp, RH	Fixed	8 s	13 months	X	✓
[57]	Temp, CO_2, VOCs, light, air velocity, acoustics	Fixed	10 min	3 years	X	X
[58]	PM1/2.5/10, NO_2, CO, O_3, SO_2, temp, RH, GPS	Fixed	5 min	4 months	X	✓
[59]	CO_2, temp, light	Fixed	N/A	1 day	X	✓
[60]	None (simulation-based)	Fixed	N/A	N/A	X	X
[61]	Temp, RH, pressure, light, altitude	Fixed	5 min	1 month	✓	X
**RoboFusion**	T (°C), H (%), pressure (hPa), altitude (m), dust (ppm), LPG, C_4H_{10}, C_3H_8, CH_4, C_2H_5OH, smoke, H_2, CO, NH_3, NO_x, aromatics, H_2S	Fixed + Portable	1 min	6 months	✓	✓

Appendix C: Implementation Transparency and Reproducibility Resources

To enhance the reproducibility and transparency of the *RoboFusion* framework, this appendix provides additional implementation details of the ESP32-based sensing nodes, communication schemas, calibration procedures, and deployment configurations. The materials presented here are designed to allow other researchers to replicate or extend the system using standard components and open-source software tools.

C.1. Firmware Structure and Pseudocode

The embedded firmware executed on each ESP32 board was designed in a modular and event-driven architecture to ensure scalability, resilience, and low power consumption. The program consists of five main routines:

1. `Acquire_Sensors()`: reads analog and digital sensor data from the 12-sensor array (temperature, humidity, pressure, dust, and six gas sensors) and stores them in memory.
2. `Update_Localization()`: reads the NFC tag identifier beneath the robot or fixed node to determine the (x, y) coordinates within the testbed.
3. `Package_Data()`: constructs a JSON packet containing sensor readings, timestamp, and suite identifier.
4. `Publish_MQTT()`: transmits the JSON packet through MQTT to the cloud broker using a secure Wi-Fi connection.
5. `Handle_Errors()`: supervises network connectivity, retries transmission if necessary, and caches unsent packets in flash memory to avoid data loss.

The firmware operates in an infinite loop with a default sampling rate of one reading per minute, configurable through a system parameter file.

Pseudocode: ESP32 Firmware Logic

```
// showing sensing, localization, packaging, MQTT publishing, and error handling.
void setup() {
  Init_WiFi();                      // Initialize Wi-Fi and MQTT client
  Init_Sensors();                   // Configure GPIO, I2C, ADC channels
  Load_Calibration_Params();        // Load offsets and scaling factors
  Serial.begin(115200);
  Connect_MQTT();                   // Establish MQTT session with broker
}
void loop() {
  // ---------- Sensor Acquisition ----------
  SensorData data;
  data.T1  = Read_DHT22_Temperature();
  data.Hum = Read_DHT22_Humidity();
  data.P   = Read_BMP280_Pressure();
  data.AL  = Compute_Altitude(data.P);
  data.T2  = Read_BMP280_Temperature();
  data.Dust = Read_GP2Y1010();
  data.MQ2 = Read_MQ(2);
  data.MQ4 = Read_MQ(4);
  data.MQ5 = Read_MQ(5);
  data.MQ7 = Read_MQ(7);
  data.MQ8 = Read_MQ(8);
  data.MQ135 = Read_MQ(135);

  // ---------- Calibration and Normalization ----------
  for each (sensor in data) {
```

```
    data[sensor] = Apply_Calibration(sensor, data[sensor]);
  }

  // ---------- Localization ----------
  NFC_Tag_ID = Read_NFC_Tag();
  Coordinates = Lookup_Coordinates(NFC_Tag_ID);

  // ---------- JSON Packaging ----------
  jsonPacket = {
     "Suite_ID": SUITE_NAME,
     "Coordinates": Coordinates,
     "Timestamp": Get_Time_ISO8601(),
     "T1": data.T1, "T2": data.T2, "H%": data.Hum, "P": data.P,
     "AL": data.AL, "Dust": data.Dust,
     "MQ2": data.MQ2, "MQ4": data.MQ4, "MQ5": data.MQ5,
     "MQ7": data.MQ7, "MQ8": data.MQ8, "MQ135": data.MQ135,
     "Condition": Current_State_Label()
  };

  // ---------- MQTT Publishing ----------
  if (WiFi_Connected() && MQTT_Connected()) {
      Publish("telemetry/" + SUITE_NAME, jsonPacket);
  } else {
      Reconnect_WiFi();
      Reconnect_MQTT();
      Cache_To_Flash(jsonPacket);  // Store unsent packets
  }

  // ---------- System Maintenance ----------
  Watchdog_Reset();
  delay(SAMPLING_INTERVAL_MS);     // Default = 60,000 ms (1 min)
}
```

The firmware acquires 12 synchronized sensor readings, associates them with a spatial coordinate through NFC localization, formats them as JSON, and transmits the data via MQTT to the ThingsBoard cloud platform. Cached retransmission and watchdog routines ensure continuous operation even during temporary communication loss.

C.2. MQTT Message Schema

Communication between the ESP32 nodes and the cloud platform is established via the Message Queuing Telemetry Transport (MQTT) protocol over port 1883, using WPA2-secured Wi-Fi connections. Each suite publishes to a unique topic named `telemetry/Suite_ID`, and the message payload follows a consistent JSON schema summarized in Table C1.

Table C1: MQTT message schema used for data publishing between ESP32 sensor suites and the ThingsBoard IoT platform.

**Field**	**Type**	**Description**
Suite_ID	String	Unique identifier of the sensor suite (e.g., "AMR1", "Suite2").
Coordinates	String	Spatial position in grid coordinates (e.g., "(5,12)").
Timestamp	ISO 8601	System timestamp of data acquisition.
T1, T2, H%, P, AL, Dust, MQ2–MQ135	Float	Sensor readings (temperature, humidity, pressure, altitude, particulate, and gas concentrations).
Condition	String	Operational state label (Normal, Hazard_1, Hazard_2, Hazard_3).

This schema ensures interoperability across all nodes and facilitates automatic data parsing and storage within the cloud platform. Each packet is typically under 300 bytes, ensuring low transmission latency and minimal bandwidth usage.

C.3. Calibration Procedures

All sensors were calibrated before deployment to ensure measurement reliability and comparability. For physical and environmental sensors (DHT-22, BMP280, GP2Y1010AU0F), factory calibration constants were validated against controlled laboratory references. For gas sensors (MQ2–MQ135), empirical calibration curves were derived using reference gas exposure tests at varying concentrations (50–1000 ppm).

Sensor analog voltage outputs (V_{out}) were converted to gas concentration C (ppm) according to the logarithmic model:

$$C = a \times \left(\frac{V_{out}}{R_o}\right)^b \tag{C1}$$

where a and b are sensor-specific constants estimated via nonlinear least-squares regression.

On-board Calibration (ESP32):

```
// 1. Preheat sensor in clean air
delay(PREHEAT_TIME);
// 2. Estimate reference resistance (Ro)
Ro = average( Rs = f(ADC_raw, RL) ) over N samples;
// 3. Store Ro in non-volatile memory
save_to_flash(sensor_id, Ro);
// 4. During operation
Rs = f(ADC_raw, RL);
ppm = 10^(((log10(Rs/Ro) - b)/c) + a);  // using {a,b,c} from calibration
```

Offline Curve-Fitting (Python):

```
# Given lab data of (ppm, Rs) and known Ro
x = log10(Rs / Ro)
y = log10(ppm)
Fit y = (x - b)/c + a    # nonlinear least-squares fit
Return calibration array {a, b, c}
```

C.4. Deployment and Configuration

Each ESP32-based sensing node communicates directly with a local Wi-Fi router, which forwards MQTT messages to the ThingsBoard Cloud platform. Configuration files define parameters such as Wi-Fi credentials, MQTT broker address, topic naming conventions, and sampling intervals. The overall system deployment and data flow architecture are illustrated in Figure C1.

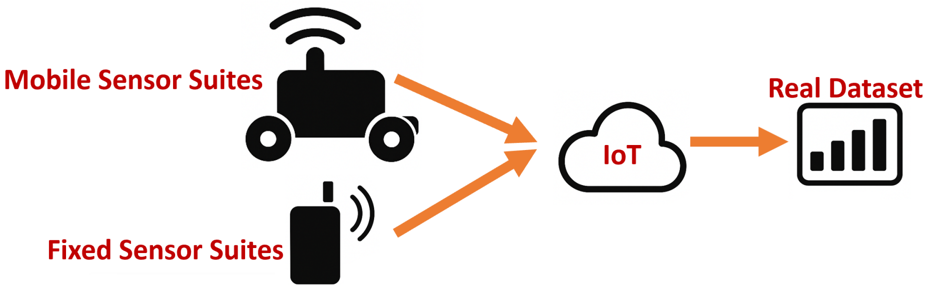

Figure C1: Deployment diagram showing mobile and fixed sensing nodes connected to the IoT platform to collect the real dataset

Appendix D: Mathematical Formulations and Model Definitions

This appendix compiles the mathematical expressions used throughout the RoboFusion framework.

Equation (D1): Pearson Correlation Coefficient

$$r = \frac{\sum_{i=1}^{n}(x_i - \bar{x})(y_i - \bar{y})}{\sqrt{\sum_{i=1}^{n}(x_i - \bar{x})^2}\,\sqrt{\sum_{i=1}^{n}(y_i - \bar{y})^2}} \tag{D1}$$

Purpose: Used to evaluate similarity between original and augmented sensor signals, confirming that augmentation does not distort temporal structure.

Parameters:

- x_i, y_i: Paired samples from two sensor sequences.
- $\bar{x}$, $\bar{y}$: Mean values of each sequence.
- n: Number of samples in the window.
- r: Linear correlation coefficient in the range $[-1, 1]$.

Equation (D2): Kullback–Leibler Divergence

$$D_{\mathrm{KL}}(P\|Q) = \sum_i P(i)\,\log\frac{P(i)}{Q(i)} \tag{D2}$$

Purpose: Measures statistical divergence between the probability distributions of real and synthetic signals, ensuring synthetic signals preserve the underlying distribution.

Parameters:

- $P(i)$: Discrete probability mass of the real dataset.
- $Q(i)$: Corresponding probability mass of the synthetic dataset.
- D_{KL}: Divergence magnitude (zero indicates identical distributions).

Equation (D3): Composite Hazard Function

$$H(t) = \begin{cases} f_{\mathrm{ign}}(t), & t_0 \le t < a, \\ f_{\mathrm{grw}}(t), & a \le t < b, \\ f_{\mathrm{peak}}(t), & b \le t < c, \\ f_{\mathrm{dec}}(t), & t \ge c, \end{cases} \tag{D3}$$

Purpose: Reconstructs a full synthetic hazard by combining four fitted segment models: Ignition, Growth, Peak, and Decay. Derived from segmentation and curve-fitting in the body text.

Parameters:

- t_0: Start time of the hazard onset.
- a, b, c: Segment boundaries identified using slope and first derivative analysis.
- $f_{\mathrm{ign}}(t)$: Ignition model (typically low-amplitude rising curve).
- $f_{\mathrm{grw}}(t)$: Growth model (rapid increase).
- $f_{\mathrm{peak}}(t)$: High-intensity plateau.
- $f_{\mathrm{dec}}(t)$: Decay model (return to baseline).

Equation (D4): Linear Normalization

$$y = y_{\min} + \frac{(x - x_{\min})(y_{\max} - y_{\min})}{x_{\max} - x_{\min}} \tag{D4}$$

Purpose: Used to ensure that each synthetic signal matches the amplitude and dynamic range of its corresponding real sensor modality.

Parameters:

- x: Original (unscaled) synthetic value.
- $x_{\min}, x_{\max}$: Minimum and maximum of the synthetic signal.
- $y_{\min}, y_{\max}$: Desired range based on real sensor data.
- y: Normalized synthetic value.

Equation (D5): Spatial Attenuation Model

$$I(d,t) = I_0 \, \frac{1}{1 + \exp\big(\alpha(d - d_0)\big)} \, S(t) \tag{D5}$$

Purpose: Models how hazard intensity weakens with distance from the origin, matching the spatial decay patterns observed in AMR and fixed-suite readings.

Parameters:

- d: Euclidean distance from hazard origin to sensor location.
- I_0: Peak hazard intensity at the source.
- α: Attenuation slope (controls steepness).
- d_0: Reference distance offset (fitting constant).
- $S(t)$: Temporal intensity profile (Equation D6).

Equation (D6): Temporal Intensity Profile (Logistic Growth)

$$S(t) = \frac{S_{\max}}{1 + \exp\big(-\gamma(t - t_0)\big)} \tag{D6}$$

Purpose: Describes how hazard intensity evolves over time, used in spatial propagation. Parameters correspond directly to the hazard type (fire, temperature fluctuations, gas leak).

Parameters:

- $S_{\max}$: Maximum temporal hazard intensity.
- γ: Temporal growth rate controlling steepness.
- t_0: Inflection point where growth transitions to saturation.

Table D1: Mathematical fit models used for each hazard segment per sensor (Ignition, Growth, Peak, Decay).

**Sensor**	**Ignition Model**	**Growth Model**	**Peak Model**	**Decay Model**
T1	Quadratic	Piecewise Linear	Gaussian	Exp Decay + Plateau
T2	Quadratic	Piecewise Linear	Gaussian	Refined Exp Decay
H%	Quadratic	Quadratic	Cubic	Cubic
Dust	Quadratic	Piecewise Linear	Gaussian	Exp Decay + Plateau
MQ2	Quadratic	Constant	Gaussian	Cubic
MQ4	Double Gaussian	Quadratic	Gaussian	Cubic
MQ5	Double Gaussian	Cubic	Gaussian	Quadratic
MQ7	Quadratic	Quadratic	Linear	Cubic
MQ8	Double Gaussian	Quadratic	Gaussian	Exp Decay
MQ135	Double Gaussian	Quadratic	Gaussian	Cubic

Appendix E: Descriptive Statistics and Model Configurations

Table E1: Overall sensor statistics across all suites - Real Dataset

sensor	count	mean	median	std	variance	min	max	range	skewness	kurtosis
T1	69120	24.52	24.4	2.4	5.78	20.4	77.6	57.2	5.19	62.48
H%	69120	52.02	52.2	7.64	58.36	11.8	71.5	59.7	-0.4	0.09
P	69120	1012.81	1012.75	3.9	15.2	1003.7	1021.45	17.75	0.0	-0.71
AL	69120	111.11	111.57	32.39	1049.36	39.52	187.01	147.49	0.01	-0.71
T2	69120	25.85	25.78	1.77	3.13	22.05	47.43	25.38	2.41	18.22
Dust	69120	0.01	0.0	0.02	0.0	0.0	0.46	0.46	3.78	34.62
MQ2	69120	59.72	11.64	2085.39	4348869.03	0.0	512627.78	512627.78	224.15	53595.4
MQ4	69120	41.97	9.37	148.85	22157.45	0.35	3613.48	3613.13	12.59	211.89
MQ5	69120	0.42	0.13	3.26	10.66	0.0	279.4	279.4	40.47	2339.63
MQ7	69120	0.03	0.0	0.33	0.11	0.0	41.05	41.05	80.11	8196.31
MQ8	69120	5.44	2.69	18.38	337.86	0.0	3056.82	3056.82	87.86	12700.35
MQ135	69120	12.45	0.3	147.23	21676.9	0.0	24558.75	24558.75	84.9	11948.31

Table E2: Per-suite sensor statistics grouped by sensor suite (complete real dataset).

Suite_ID	sensor	count	mean	median	std	variance	min	max	range	skewness	kurtosis
AMR_1	T1	17280	24.42	24.3	2.38	5.67	21.2	67.2	46.0	6.61	80.14
AMR_1	H%	17280	51.21	52.3	8.68	75.29	11.8	68.93	57.13	-0.47	0.05
AMR_1	P	17280	1012.53	1012.56	3.89	15.1	1003.91	1020.57	16.66	0.0	-0.73
AMR_1	AL	17280	113.42	113.14	32.29	1042.92	46.78	185.32	138.54	0.01	-0.73
AMR_1	T2	17280	25.36	25.37	1.78	3.17	22.05	39.94	17.89	1.6	9.74
AMR_1	Dust	17280	0.0	0.0	0.0	0.0	0.0	0.02	0.02	58.17	3796.94
AMR_1	MQ2	17280	0.1	0.06	0.32	0.1	0.0	15.49	15.49	24.05	843.46
AMR_1	MQ4	17280	3.64	2.95	4.49	20.14	0.35	165.13	164.78	10.88	216.67
AMR_1	MQ5	17280	0.46	0.01	1.18	1.4	0.0	38.61	38.61	7.32	143.57
AMR_1	MQ7	17280	0.0	0.0	0.0	0.0	0.0	0.01	0.01	131.44	17275.0
AMR_1	MQ8	17280	2.59	2.36	1.89	3.58	0.0	63.0	63.0	8.78	141.75
AMR_1	MQ135	17280	0.0	0.0	0.04	0.0	0.0	2.27	2.27	40.9	1933.34
AMR_2	T1	17280	23.08	22.9	2.52	6.37	20.4	77.6	57.2	8.86	120.03
AMR_2	H%	17280	54.7	55.5	6.68	44.59	17.2	67.1	49.9	-0.52	0.54
AMR_2	P	17280	1013.08	1013.1	3.87	15.0	1004.47	1021.16	16.69	0.0	-0.73
AMR_2	AL	17280	108.92	108.69	32.17	1035.0	41.89	180.59	138.7	0.01	-0.73
AMR_2	T2	17280	25.53	25.46	1.54	2.39	22.78	40.65	17.87	2.9	21.07
AMR_2	Dust	17280	0.0	0.0	0.01	0.0	0.0	0.24	0.24	17.06	314.36
AMR_2	MQ2	17280	154.96	60.74	4157.52	17284K	16.48	512K	512K	113.08	13K
AMR_2	MQ4	17280	11.58	9.44	18.29	334.69	4.4	1309.21	1304.81	32.03	1762.4
AMR_2	MQ5	17280	0.57	0.18	6.07	36.9	0.0	279.4	279.4	24.22	768.21
AMR_2	MQ7	17280	0.08	0.04	0.62	0.38	0.0	41.05	41.05	45.28	2523.32
AMR_2	MQ8	17280	13.86	10.26	34.73	1206.05	3.09	3056.82	3053.73	51.34	3934.77
AMR_2	MQ135	17280	39.33	13.67	254.73	64889.8	1.24	24558.75	24.5K	58.11	5111.9
Suite_1	T1	17280	25.47	25.4	2.04	4.16	21.6	57.2	35.6	3.77	34.12
Suite_1	H%	17280	49.98	50.1	6.28	39.39	25.1	67.4	42.3	-0.28	-0.33
Suite_1	P	17280	1013.31	1013.33	3.88	15.03	1004.73	1021.45	16.72	0.0	-0.73
Suite_1	AL	17280	106.94	106.76	32.2	1036.89	39.52	178.44	138.92	0.01	-0.73
Suite_1	T2	17280	26.51	26.39	1.73	2.98	22.98	45.22	22.24	2.9	21.66
Suite_1	Dust	17280	0.01	0.0	0.02	0.0	0.0	0.25	0.25	6.88	54.28
Suite_1	MQ2	17280	0.0	0.0	0.01	0.0	0.0	1.08	1.08	84.62	8087.41
Suite_1	MQ4	17280	139.97	55.55	273.0	74.5K	2.95	3613.48	3610.53	7.02	62.0
Suite_1	MQ5	17280	0.01	0.0	0.04	0.0	0.0	1.76	1.76	16.12	420.66
Suite_1	MQ7	17280	0.0	0.0	0.0	0.0	0.0	0.01	0.01	131.44	17275.0
Suite_1	MQ8	17280	5.28	3.61	5.78	33.4	0.58	182.74	182.16	8.48	145.8
Suite_1	MQ135	17280	0.01	0.0	0.14	0.02	0.0	4.61	4.61	16.09	299.97
Suite_2	T1	17280	25.11	25.0	1.9	3.59	22.4	64.4	42.0	5.48	72.27
Suite_2	H%	17280	52.21	52.7	7.88	62.16	17.4	71.5	54.1	-0.31	-0.26
Suite_2	P	17280	1012.33	1012.34	3.88	15.04	1003.7	1020.34	16.64	0.0	-0.73

Continued on next page

Table E2 – continued from previous page

Suite_ID	sensor	count	mean	median	std	variance	min	max	range	skewness	kurtosis
Suite_2	AL	17280	115.15	114.95	32.23	1038.95	48.7	187.01	138.31	0.01	-0.73
Suite_2	T2	17280	26.01	25.9	1.78	3.18	23.1	47.43	24.33	3.21	28.13
Suite_2	Dust	17280	0.04	0.04	0.01	0.0	0.0	0.46	0.46	23.36	741.23
Suite_2	MQ2	17280	83.83	43.33	307.43	94.51K	0.0	10.87K	10.87K	19.86	516.0
Suite_2	MQ4	17280	12.69	8.6	29.93	895.56	0.6	1500.74	1500.14	19.47	622.69
Suite_2	MQ5	17280	0.62	0.38	2.02	4.1	0.0	93.26	93.26	20.61	624.39
Suite_2	MQ7	17280	0.02	0.01	0.21	0.04	0.0	17.97	17.97	60.39	4369.35
Suite_2	MQ8	17280	0.02	0.01	0.21	0.04	0.0	17.97	17.97	60.39	4369.35
Suite_2	MQ135	17280	10.44	1.13	144.17	20.78K	0.0	8200.0	8200.0	39.47	1907.04

Table E3: Curve-fit parameters for all sensors (complete sensor-wise coefficients, RMSE and R^2).

Sensor	Stage	Coefficients	RMSE	R²
T1	Ignition	[2.31942027e-04, -4.69698254e-01, 2.62511748e+02]	0.11	0.06
T1	Growth	[4.00499992e+00, -4.11180991e+03, 1.87500000e+00, -1.89956667e+03, 1.04000002e+03]	0.45	1.0
T1	Peak	[77.97991588, 1050.64273745, 6.50333638]	0.0	1.0
T1	Decay	[-8.40870312e+08, 1.32269709e+05, -6.74349276e-01, 7.67886932e+02]	4.6	0.87
T2	Ignition	[-1.07849961e-03, 2.19891448e+00, -1.09418072e+03]	0.01	0.97
T2	Growth	[4.40000000e-01, -4.28193333e+02, 3.97500108e-01, -3.83640113e+02, 1.04000000e+03]	0.09	1.0
T2	Peak	[35.34760993, 1051.7765048, 10.77009133]	0.0	1.0
T2	Decay	[3.26470325e+03, 4.24391693e-04, 7.31889184e-01, -2.82211520e+03]	0.45	0.97
H%	Ignition	[1.93782321e-03, -3.96814019e+00, 2.08864266e+03]	0.15	0.71
H%	Growth	[2.31547619e-01, -4.83676190e+02, 2.52608100e+05]	1.01	0.99
H%	Peak	[1.875000e-01, -3.941250e+02, 2.071297e+05]	0.0	1.0
H%	Decay	[-4.31546082e-04, 1.39579177e+00, -1.50392051e+03, 5.39844972e+05]	1.45	0.99
Dust	Ignition	[0.00e+000, -4.94e-323, 2.44e-320]	0.0	1.0
Dust	Growth	[1.81365685e-14, -1.87031625e-11, -9.89225047e-15, 1.03395521e-11, 1.04000000e+03]	0.0	0.0
Dust	Peak	[0.0, 1050.0, 10.0]	0.0	1.0
Dust	Decay	[4.44935954e+01, 1.73125325e-05, 5.58935327e+04, -1.14845758e+02]	0.068	0.205
MQ2	Ignition	[-1.79007757e-02, 1.02501694e+02, -8.51101214e+04]	1061.35	0.22
MQ2	Growth	[244.16714286]	60.7	0.0
MQ2	Peak	[1143.22742778, 1052.81756733, 8.28150121]	0.0	1.0
MQ2	Decay	[8.86101982e-02, -2.88445133e+02, 3.12907349e+05, -1.13119629e+08]	1053.53	0.25
MQ4	Ignition	[770.261952, 18.68859955, 1.12889502]	108.4	0.633
MQ4	Growth	[8.07738095e-01, -6.28407143e+01, 1.25403476e+03]	5.92	0.932
MQ4	Peak	[63.44377573, 0.6501211, 48.211468, 21.33524364]	5.29	0.562
MQ4	Decay	[3.34958690e-03, -8.57542290e-01, 6.96473744e+01, -1.67745630e+03]	104.77	0.084
MQ5	Ignition	[9.67716721e-01, 4.90696553e-01, 1.02423912e+03, 4.58216502e+01]	11.57	0.21
MQ5	Growth	[-4.88636787e-01, 1.52447131e+03, -1.58535820e+06, 5.49554326e+08]	15.46	0.69
MQ5	Peak	[20.59894015, 1051.53029002, -1.93352447]	0.0	1.0
MQ5	Decay	[5.53491975e-03, -1.21027396e+01, 6.61577756e+03]	2.8	0.44
MQ7	Ignition	[-5.13534260e-03, 1.11924861e+01, -6.06387863e+03]	9.6	0.31
MQ7	Growth	[4.17857143e-02, -8.72139286e+01, 4.55087414e+04]	0.28	0.96
MQ7	Peak	[1.7175e+00, -1.799945e+03]	1.75	0.72
MQ7	Decay	[1.16437731e-04, -3.79951203e-01, 4.13118097e+02, -1.49664706e+05]	6.24	0.07
MQ8	Ignition	[1.45191083, 2.00204402, 1.02199931, 6.50771884]	90.65	-0.329
MQ8	Growth	[0.19227679, -2.86516073, 31.07300004]	5.32	0.566
MQ8	Peak	[344.73734345, 1.62991528, 3.88792205, 54.47102042]	0.0	1.0

Continued on next page

Table E3 – continued from previous page

Sensor	Stage	Coefficients	RMSE	R^2
MQ8	Decay	[5.56384899e+01, 8.92220475e-01, 7.34724775e+02, 3.81814301e-04, -6.76548252e+02]	113.62	-0.094
MQ135	Ignition	[-8.72549454e+05, -2.48863688e+02, -2.37181734e+00, 1.47864826e+58, 1.84506413e+02, 9.86485290e+00]	46.26	0.997
MQ135	Growth	[6.13395824, -102.39232023, 439.79523581]	51.76	0.884
MQ135	Peak	[1034.11946128, 2.31445611, 3.97812671, 73.16505002]	2.94	1.0
MQ135	Decay	[1.64701556e+03, 2.37130727e-02, 1.82897832e+03, 2.37260454e-02, -8.22526220e+02]	1803.43	0.147

Table E4: Key hyperparameters of the ML models used across the three evaluation scenarios.

Model	Parameter	S1	S2	S3	Notes
**Random Forest**					
	n_estimators	200	200	200	Balanced accuracy; stable learning.
	max_depth	8	8	10	Slightly deeper for cross-domain.
	min_samples_split	2	2	2	Default; avoids under-splitting.
	max_features	sqrt	sqrt	sqrt	Ensures feature diversity.
	bootstrap	True	True	True	Bagging enabled.
**XGBoost**					
	n_estimators	300	300	350	Slight increase in S3 for transfer.
	learning_rate (eta)	0.10	0.10	0.05	Slower learning improves cross-domain generalization.
	max_depth	6	6	7	Moderate regularization.
	subsample	0.8	0.8	0.8	Row sampling.
	colsample_bytree	0.8	0.8	0.8	Feature sampling.
	lambda (L2)	1	1	1	Default regularization.
	alpha (L1)	0	0	0	Default.
**SVM (RBF)**					
	C	–	1.0	1.0	Default soft-margin regularization.
	kernel	–	rbf	rbf	Radial kernel.
	gamma	–	0.01	0.01	Tuned via grid search.
	class_weight	–	balanced	balanced	To handle data imbalance.
**MLP**					
	hidden_layer_sizes	–	(64, 32)	(64, 32)	Two-layer feed-forward.
	activation	–	relu	relu	Non-linear mapping.
	solver	–	adam	adam	Adaptive optimizer.
	learning_rate_init	–	0.001	0.001	Stable training.
	alpha (L2)	–	0.0001	0.0001	Mild weight decay.
	max_iter	–	500	500	Convergent training.
